# High-salt diet mediates interplay between NK cells and gut microbiota to induce potent tumor immunity

**DOI:** 10.1126/sciadv.abg5016

**Published:** 2021-09-10

**Authors:** Zaigham Abbas Rizvi, Rajdeep Dalal, Srikanth Sadhu, Yashwant Kumar, Shakti Kumar, Sonu Kumar Gupta, Manas Ranjan Tripathy, Deepak Kumar Rathore, Amit Awasthi

**Affiliations:** 1 Immunbiology Lab, Translational Health Science and Technology Institute, NCR-Biotech Science Cluster, 3rd Milestone, Faridabad-Gurgaon Expressway, Faridabad, Haryana 121001, India.; 2 Infection and Immunology, Translational Health Science and Technology Institute, NCR-Biotech Science Cluster, 3rd Milestone, Faridabad-Gurgaon Expressway, Faridabad, Haryana 121001, India.; 3 Noncommunicable Disease Center, Translational Health Science and Technology Institute, NCR-Biotech Science Cluster, 3rd Milestone, Faridabad-Gurgaon Expressway, Faridabad, Haryana 121001, India.

## Abstract

High-salt diet (HSD) modulates effector and regulatory T cell functions and promotes tissue inflammation in autoimmune diseases. However, effects of HSD and its association with gut microbiota in tumor immunity remain undefined. Here, we report that HSD induces natural killer (NK) cell–mediated tumor immunity by inhibiting PD-1 expression while enhancing IFNγ and serum hippurate. Salt enhanced tumor immunity when combined with a suboptimal dose of anti-PD1 antibody. While HSD-induced tumor immunity was blunted upon gut microbiota depletion, fecal microbiota transplantation (FMT) from HSD mice restored the tumor immunity associated with NK cell functions. HSD increased the abundance of *Bifidobacterium* and caused increased gut permeability leading to intratumor localization of *Bifidobacterium*, which enhanced NK cell functions and tumor regression. Intratumoral injections of *Bifidobacterium* activated NK cells, which inhibited tumor growth. These results indicate that HSD modulates gut microbiome that induces NK cell–dependent tumor immunity with a potential translational perspective.

## Teaser

Salt triggers tumor immunity through microbiota-mediated NK cell activation.

## INTRODUCTION

Dietary components influence human health by regulating immune homeostasis and gut microbiota composition (*1*–*4*). Salt when taken in a higher amount [4% NaCl: high-salt diet (HSD)] has been identified as a potent immunomodulator associated with a strong inflammatory response (*5*–*7*). Recent studies identified that HSD exacerbates tissue inflammation in ulcerative colitis and autoimmune encephalomyelitis and increases the risk of cardiovascular diseases associated with enhanced T helper 17 (T_H_17) cell development and functions (*5*, *8*, *9*). Other studies reported that HSD polarizes macrophages to M1-like phenotype and its association with elevated interferon-γ (IFNγ) response (*6*, *7*, *10*). A longitudinal study on healthy human participants found a strong correlation between HSD and monocyte frequency (*6*). On the basis of these observations, HSD could act as an inflammatory trigger that may overcome immunosuppressive conditions associated with tumor microenvironment such as the expression of checkpoint inhibitors and down-regulation of major histocompatibility complex I (MHC-I) molecules. Recent studies have shown that HSD can inhibit tumor growth, which may be dependent on myeloid-derived suppressor cells (MDSCs) (*11*, *12*).

Down-regulation of MHC-I is a strong activation signal for NK cell activation and mediates direct killing of tumor cells (*13*). Activation of NK cells is in turn controlled by a wide array of activation signals such as CD107a, natural cytotoxic trigger receptor 1 (NCR1), CD226, and inhibitory signals such as CD96, programmed cell death protein (PD) 1, T cell immunoglobulin and ITIM domain (TIGIT), T-cell immunoglobulin domain and mucin domain (Tim) 3, and cytotoxic T-lymphocyte associated protein (CTLA) 4 molecules (*14*–*17*). Furthermore, the tumor microenvironment is often characterized by ionic imbalance as decreased sodium level (hyponatremia) has been linked to human cancers (*18*, *19*). Altered Na^+^/H^+^ concentration across the gut epithelial barrier is linked with changes in gut permeability and dysbiosis, and previous reports have suggested that HSD induces changes in the gut microbiota composition and metabolic alterations in rodents (*20*). These shreds of evidence suggested that tumor immunity by HSD may involve factors from serum and gut microbiota and may influence other components of the immune system essential for antitumor functions.

In the current study, we report that tumor-bearing mice fed with HSD potently suppressed tumor growth by up-regulation of NK cell frequency and activation markers and down-regulation of NK cell inhibitory signals (especially PD1 molecule). NK cell depletion truncated the tumor immunity of HSD, which was found to be mediated by NK-dependent interferon-γ (IFNγ) response. We further establish that HSD, in mice, leads to marked up-regulation of serum hippurate, a microbial benzoate metabolism product that is also described as one of the metabolic markers of PD-1 immunotherapy in responding patients (*21*). In line with this, we report that the combination of a suboptimal dose of anti-PD1 antibody together with a low-salt diet provides a significant tumor regression. Antibiotic-induced gut microbiota depletion (AIMD) abrogated the HSD-mediated tumor inhibition and antitumor NK cell functions, indicating the involvement of gut microbiota. HSD-fed mice showed an increased abundance of *Bifidobacterium* in their stool, which upon transfer to AIMD mice, suppressed tumor progression associated with increased intratumor NK cell frequency and elevated serum hippurate levels. Last, we demonstrate that mice fed with HSD show an increased gut permeability resulted in intratumoral localization of *Bifidobacterium* leading to NK cell activation. While intratumoral administration of *Bifidobacterium* alone resulted in tumor regression, NK cell depletion blunted *Bifidobacterium*-mediated protection. Furthermore, increased hippurate levels were found in *Bifidobacterium* administered mice suggesting that hippurate might be a potential biomarker of HSD-mediated tumor immunity. Together, we provide the mechanistic insight into the factors involved in HSD-mediated tumor immunity and show that *Bifidobacterium* and NK cross-talk is essential in mounting tumor immunity. Our preclinical data strongly suggest the therapeutic potential of HSD and points to its potential translational application.

## RESULTS

### HSD imparts robust tumor immunity in syngeneic mouse tumor models

**Fig. 1.**
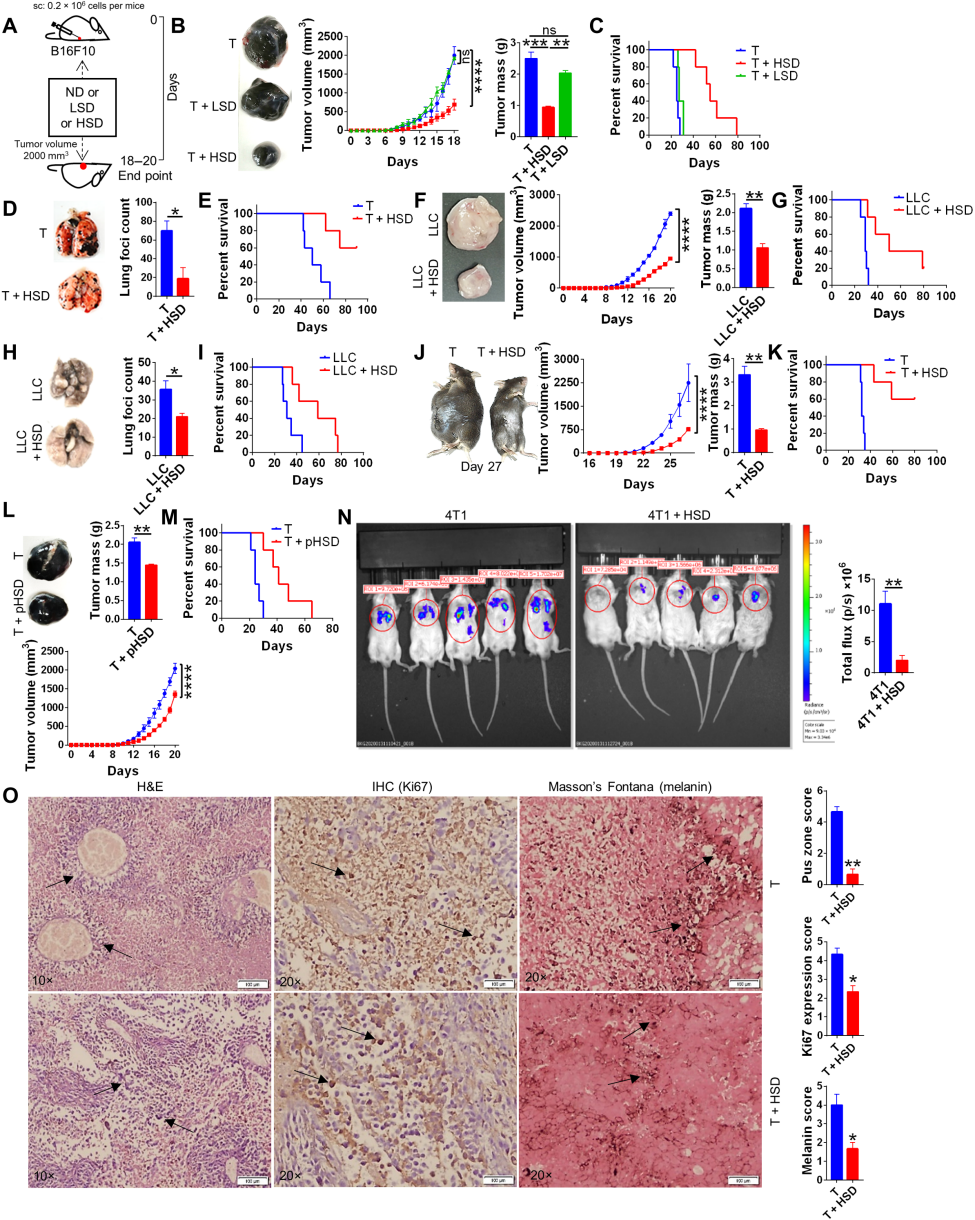
HSD provides potent immunity against murine syngenic tumor. Syngenic mouse tumor models by using subcutaneous (melanoma) or intravenous (metastasis) injections of cell lines was used to study the effect of normal salt diet (T + 0.9% NaCl), low-salt diet [T + LSD (1% NaCl above ND)], or high-salt diet [T + HSD (4% NaCl above ND)] until tumor volume reached 2000 mm^3^ or until animal mortality for survival curve. At the end point (when tumor volume reached ~2000 mm^3^), the animals were euthanized; their tumor was excised, cleaned, and imaged; and their tumor mass was recorded. For metastasis model, animals were euthanized 20 days after intravenous injections, and their lung was excised to count the number of lung foci. (**A**) Schematic representation of the method for (**B**) progression of B16 skin melanoma with different doses of salt diet and (**C**) survival curve of B16 melanoma animals. ns, not significant; sc, subcutaneous. B16 lung metastasis model (**D**) showing foci in lungs and (**E**) survival curve. (**F** and **G**) Carcinoma and (**H** and **I**) lung metastasis with their (G and I) survival curve, respectively, by LLC cells and (**J**) surgical model in which tumor from mice was surgically removed at 1000 mm^3^, and then the animals were put either on HSD or ND and its (**K**) survival curve. (**L**) Pretreatment model in which one group of animal was pretreated for 15 days with HSD, and then B16 melanoma was allowed to grow with ND and its (**M**) survival curve. (**N**) Lung metastasis of 4T1 luciferase cells on day 16 by in vivo imaging of luminescence. (**O**) Histological images of B16 melanoma on day 15: hematoxylin and eosin (H&E) stain at 10× (left), immunohistochemistry (IHC) for Ki67 at 20× (middle), and Masson’s Fontana stain at 20× (right). *P < 0.05, **P < 0.01, ***P < 0.001, and ****P < 0.0001 (Student’s *t* test or one-way ANOVA). Photo credit: Z. Abbas Rizvi, Translational Health Science and Technology Institute.

As a proof of concept, we studied the effect of salt on B16F10 skin melanoma (abbreviated B16) in mice. For this, mice implanted with B16 cells subcutaneously were simultaneously fed with three different doses of dietary salt viz. normal diet (ND) containing naturally occurring 0.9% NaCl above ND, low-salt diet (LSD) containing 1.0% NaCl above ND, and HSD consisting of 4% NaCl above ND as shown in Fig. 1A. We found that as compared to ND, mice fed with HSD significantly reduced tumor progression measured by tumor volume and mass associated with an overall increased percent survival (Fig. 1, B and C). No significant tumor regression and percent survival were noticed in the mice fed with LSD (Fig. 1, B and C). We did not observe any significant changes in food and water intake in mice of tumor (T), T + LSD, and T + HSD groups (fig. S1A), indicating that the salt tumor immunity induced in HSD is not through calorie restrictions. Since low sodium (Na^+^) serum levels and ionic imbalances have been reported in patients with certain cancers (*19*), we hypothesized that HSD intake may restore serum Na^+^ levels and mitigate the effects of hyponatremia. In line with this, we found around 35% reduction in the serum Na^+^ level at end point in the mice with melanoma as compared to healthy (H) control, which was restored to normal levels in mice fed with HSD (fig. S1B).

There were no changes observed in serum potassium (K^+^), serum glutamic pyruvic transaminase, serum glutamic oxaloacetic transaminase, alkaline phosphatase, creatinine, urea, and uric acid levels in either T or T + HSD group (fig. S1B and S1C). Since increased salt uptake is often associated with an increased risk of cardiovascular complications and osmolarity-induced histological changes in healthy individuals, we evaluated the risk of physiological and organ-specific damages associated with HSD-fed tumor-bearing mice. Unexpectedly, our data suggest that HSD is well tolerated without any toxicological effects on major organs such as the brain, colon, heart, kidneys, liver, lungs, and spleen with no gross histological changes and no obvious signs of arrhythmia (evaluated through electrocardiogram) between H and T + HSD groups (fig. S1, D and E). It was previously reported that dietary intake of HSD could attenuate metastasis through hyperosmotic stress via p53 (*22*). On contrary, we found that mannitol, when used at equi-iso-osmolar to 4.0% NaCl, failed to regress B16 melanoma and improve survival (fig. S1, F and G). Next, we investigated the protective efficacy of HSD on tumors of different origins and found that HSD-fed mice imparts robust tumor immunity against B16 lung metastasis that showed ~35% decrease in lung foci count; Lewis lung carcinoma (LLC), giving twofold inhibition of tumor growth and mass; LLC lung metastasis showing around 1.75-fold decrease in lung foci count; HSD-treated mice after surgical removal of B16 melanoma imparted around threefold reduction in tumor growth and mass; and reduced tumor progression in mice given pretreatment of HSD for 15 days and then kept on ND after B16 injections [HSD pretreatment (pHSD)], with overall improved percent survival rate across all tumor models (Fig. 1, D to M, respectively). By using 4T1 (breast cancer cell line) luciferase cell line to study lung metastasis, it was found that HSD limits seeding of 4T1 not only in lungs but also in other organs with around sixfold reduction in total flux emission (Fig. 1N). Our histological data revealed that HSD-induced regression in B16 melanoma was characterized by reduced “pus” zones, tumor cell proliferation (Ki67 expression), and melanin pigmentation in HSD group (Fig. 1O). The control Ki67 samples without anti-Ki67 antibody showed no immunohistochemistry staining (fig. S1H). Together, these data demonstrate that HSD imparts robust tumor immunity without noticeable side effects on normal physiology.

### NK cell–driven IFNγ is essential for HSD-mediated tumor immunity

**Fig. 2.**
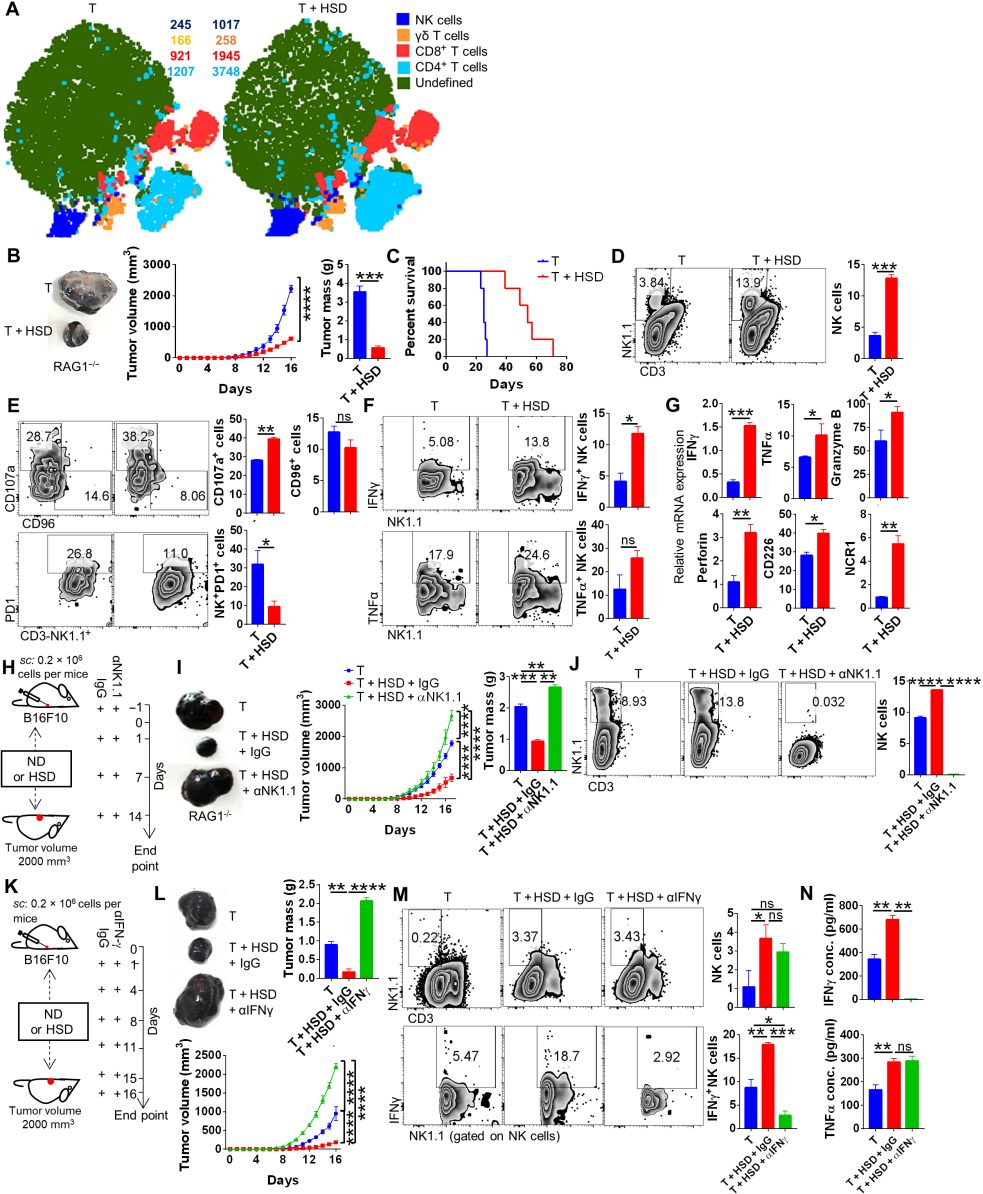
HSD tumor immunity is mediated by elevated NK cell frequency and activation. Tumor-infiltrating immune cells isolated from B16 melanoma were profiled for immune cell population through flow cytometry after excising the tumor at the end point. The results from immunophenotyping was then analyzed by using graph pad and was plotted as bar graphs representing mean % positive cells ± SEM along with representative FACS zebra plot and (**A**) representative t-distributed stochastic neighbor embedding (tSNE) plot depicting clusters and counts of NK, γδT, CD4, and CD8 cells. (**B**) B16 melanoma progression in RAG1^−/−^ mice and its (**C**) survival cure. (**D** to **F**) Immunophenotyping for NK cells; expression of CD107a, CD96, and PD1 molecule on NK cells; and expression of IFNγ and TNFα by NK cells. (**G**) Relative mRNA expression of NK cell–associated genes from tumor-infiltrating immune cells samples. (**H**) Schematic representation for (**I**) progression of B16 melanoma in the presence of HSD with or without NK cell depletion and (**J**) its profiling for NK cells. (**K**) Schematic representation for the effect of neutralization of IFNγ on (**L**) B16 melanoma progression and (**M**) changes in NK cells and IFNγ expressed by NK cells. (**N**) Levels of serum cytokines evaluated through ELISA. *P < 0.05, **P < 0.01, ***P < 0.001, and ****P < 0.0001 (Student’s *t* test or one-way ANOVA).

To understand the changes in the immunological response of ND and LSD versus HSD-fed melanoma-bearing C57BL/6 mice, we carried out extensive immune cell profiling of tumor-infiltrating immune cells, draining lymph nodes, and splenocytes. Our data identified ~50% up-regulation of NK cell frequency and its activation marker, CD107a, along with inhibition of PD1 expression in mice fed with HSD, while no significant changes were observed in mice fed with either LSD or ND (fig. S2, A and B). Moreover, mice fed with HSD, as compared to ND or LSD, showed less prominent changes in the frequencies of other major immune cell populations viz. CD4^+^ and CD8^+^ T cells, γδT cells, macrophages, and MDSCs of tumor-infiltrating immune cells (Fig. 2A and fig. S2, C and D). In addition, the expression of other inhibitory markers, Tim3 and TIGIT, remain unchanged, though significant down-regulation of CTLA4 was observed on NK cells (fig. S2E). Expression of IFNγ in NK cells was found to be significantly up-regulated, while tumor necrosis factor–α (TNFα) and interleukin-17A (IL-17A) levels remained the same in the melanoma-bearing mice fed with HSD as compared to ND (fig. S2F). Next, to validate that HSD-mediated tumor immunity is independent of adaptive immunity (B cell, CD4, and CD8 cells), we implanted B16 melanoma cells in recombination activating gene 1 knockout (RAG1^−/−^) mice, as these mice lack mature T and B cells without affecting the populations of innate cells including NK cells.

As compared to ND, B16-bearing RAG1^−/−^ mice fed with HSD showed ~4-fold reduction in tumor volume and ~7-fold reduction in tumor mass associated with improved survival (Fig. 2, B and C), indicating that HSD-mediated tumor immunity is independent of mature T and B cells and potentially rely upon innate immune cells. Further, flow cytometry analysis reveals that, as compared to ND, B16-bearing HSD-fed RAG1^−/−^ mice show a higher frequency of NK cells in tumor-infiltrating immune cells, which was accompanied by increased CD107a expression and lowered PD1 expression on NK cells (Fig. 2, D and E). Consistently, tumor-infiltrating immune cells of B16-bearing HSD-fed RAG1^−/−^ mice showed a significant up-regulation in IFNγ and a marginal increase in TNFα levels in NK cells (Fig. 2F). In addition, the mRNA expression of PDL1 in melanoma cells was significantly decreased in B16-bearing mice kept on HSD as compared to mice kept on ND (fig. S2G). Moreover, mRNA expression of effector molecules of NK cells from tumor-infiltrating immune cells from B16-bearing HSD-fed RAG1^−/−^ mice, as compared to B16-bearing ND-fed RAG1^−/−^ mice, showed a robust increase in cytokines (IFNγ and TNFα), cytotoxic factors (perforin and granzyme B), and receptors (CD226 and NCR1) (Fig. 2G). To further ascertain the role of NK cells in tumor immunity induced by HSD, we depleted NK cells using anti-mouse NK1.1 antibody in HSD-fed B16-bearing RAG1^−/−^ mice (Fig. 2H). The depletion of NK cells blunted the protective effect of HSD on B16 growth in RAG1^−/−^ mice as measured by tumor volume and mass (Fig. 2I). Anti-NK1.1 antibody efficiently depleted NK cell population (Fig. 2J). Furthermore, since IFNγ secreted from NK cells plays an important role in tumor immunity (*23*) and was found to be produced by NK cells in HSD-fed mice, we next investigated whether NK cell–driven IFNγ is critical in tumor immunity induced by HSD. Our data show that HSD suppressed B16 tumor growth in mice and is associated with a higher frequency of NK cells and NK cell–driven IFNγ production from tumor-infiltrating immune cells, while IFNγ neutralization abrogated the protective effect of HSD on tumor growth (Fig. 2, K and L) with effective neutralization of serum IFNγ but not TNFα (Fig. 2, M and N). Further, the mRNA expression of cytotoxic markers on sorted NK cell population from tumor-infiltrating immune cells showed significant up-regulation in the presence of HSD (fig. S2H). Together, these data demonstrated that HSD-induced tumor immunity is mediated by NK cell and IFNγ production.

### Salt induces metabolomics changes in serum with elevated serum hippurate levels

**Fig. 3.**
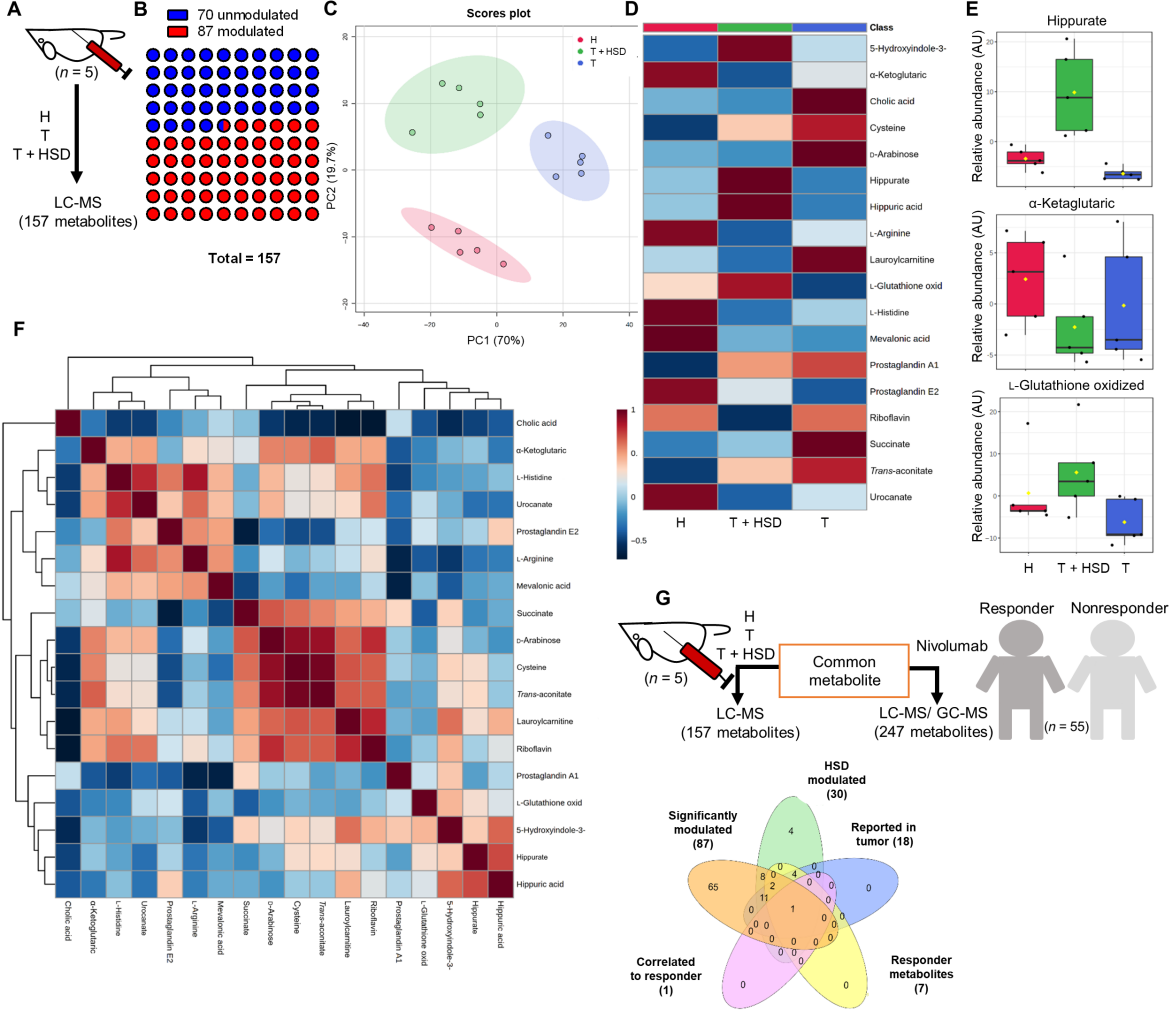
Serum metabolite profiling of HSD-fed B16 melanoma-bearing mice. Serum of the salt-fed mice were profiled through LC-MS to understand the metabolomics changes associated with HSD fed B16 melanoma-bearing mice. (**A**) Schematic representation for the methodology and number of identified metabolites. (**B**) Number of significantly modified metabolites identified through ANOVA. (**C**) PCA distribution of the metabolomics profile from different groups. (**D**) Average heatmap for top 17 metabolites. (**E**) Normalized abundance levels of three metabolites was plotted. AU, arbitrary unit. (**F**) Correlation of identified metabolites with each other and (**G**) with the metabolomics profile of responder patients for anti-PD1 therapy was overlaid to identify common metabolite.

Anti-PD1 antibody–mediated therapy for various tumors has shown great success in patients with cancer and has been recently found to be associated with the up-regulation of certain metabolites in responder (R) groups as compared to nonresponder (NR) groups (*21*, *24*). On the similar line, we set out to understand what metabolomic changes occur in the serum of tumor-bearing mice fed with HSD. To understand this, we compared metabolomics changes associated with H, T, and T + HSD groups in mice. Our liquid chromatography–tandem mass spectrometry (LC-MS/MS) data identified that 87 of 157 total metabolites were significantly modulated (Fig. 3, A and B). The principal components analysis (PCA) plot of H, T, and T + HSD showed a distinct distribution pattern of identified metabolites, indicating that the three groups have unique metabolomics profiles (Fig. 3C). There were changes in 87 modulated metabolites, and the heatmap indicated that HSD metabolomics profile was similar to H group (fig. S3A). Our metabolomics results indicate prominent changes in hippurate, 5-hydroxyindole acetic acid, cholic acid, α-ketoglutarate, l-glutathione oxidized, urocanate, etc. in B16-bearing mice fed with HSD (Fig. 3, D and E, and fig. S3B), which showed good correlation with each other (Fig. 3F and fig. S3C). Further analysis revealed that pathways related to amino acid metabolism, mevalonate-aspartate pathway, and Warburg effect were perturbed (fig. S3D). We compared serum metabolites profile of T + HSD group with previously published reports on HSD serum metabolites, metabolic markers of published cancer therapeutics. In addition, a recent study by Honjo group had identified serum metabolites of patients that responded well to anti-PD1 cancer immunotherapy (*21*). To investigate a correlation between the above mentioned groups serum metabolite perturbed by HSD and serum profile of anti-PD1 therapy of the R group, we generated an interactive Venn diagram by literature survey of previously published reports. Of the 87 significantly modulated metabolites in our study, 30 metabolites were found to be common between our study and previously reported HSD serum metabolites profile, 18 metabolites were found to be common in our study and previously published literature on markers for cancer therapeutics, 7 metabolites were found to be common between our study and recently published study of responders of anti-PD1 cancer-therapy in human with the R metabolite profile, and 1 metabolite, hippurate, was uniquely common in all the groups as indicated (Fig. 3G). Taken together, these results indicated changes in serum metabolites associated with T + HSD in which hippurate was found to be increased by HSD.

### Salt enhanced anti-PD1 therapy and tumor regression

**Fig. 4.**
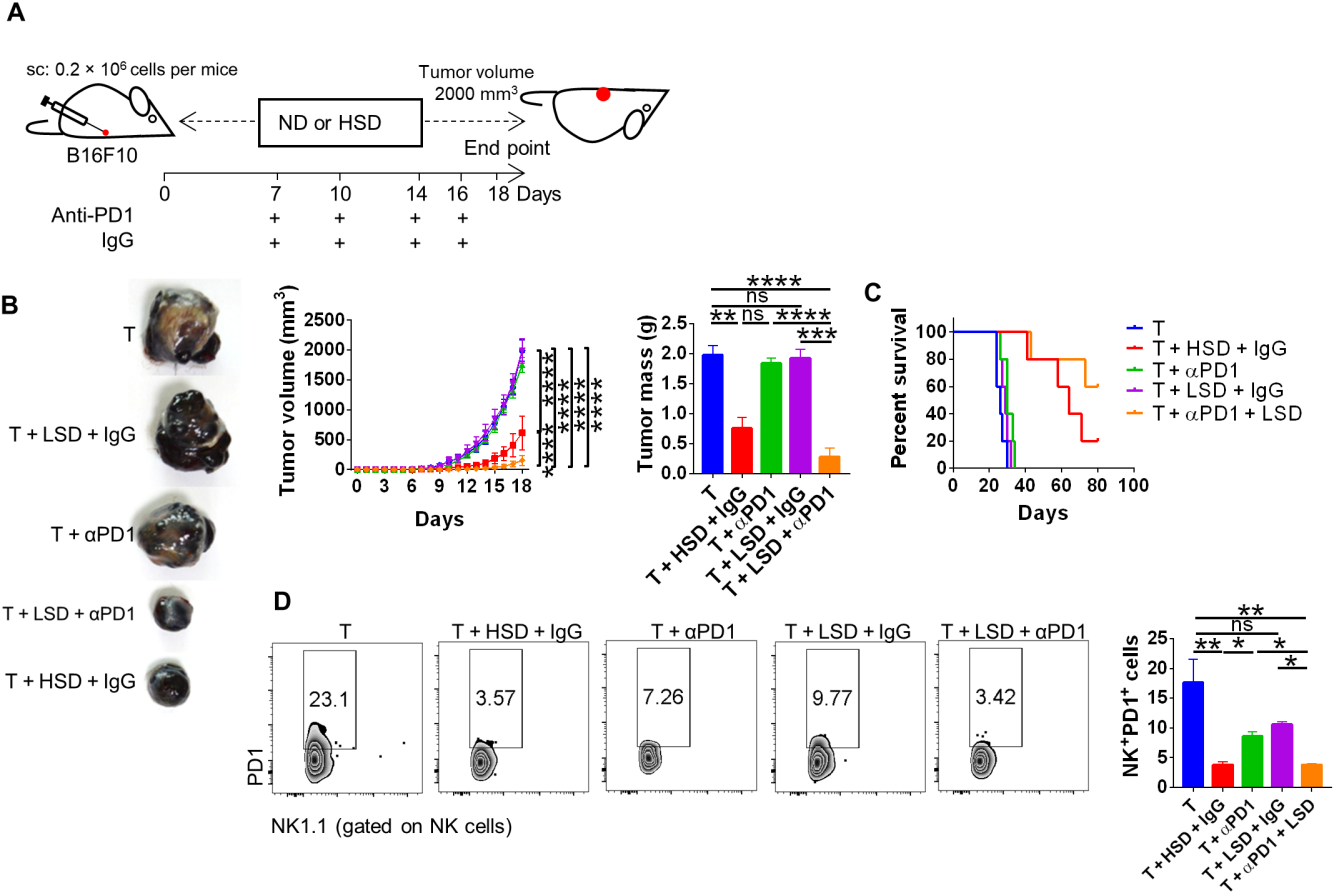
Use of salt as an adjuvant in combination with suboptimal dose of anti-PD1–neutralizing antibody. (**A**) Schematic methodology and (**B**) tumor progression for combinatorial therapy involving salt and suboptimal dose of anti-PD1–neutralizing antibody and (**C**) its survival curve and (**D**) the subsequent profiling for PD1 expression on NK cells. *P < 0.05, **P < 0.01, ***P < 0.001, and ****P < 0.0001 (Student’s *t* test or one-way ANOVA).

Since hippurate had already been identified as potent biomarker of R group of anti-PD1 therapy from the clinical patients, we hypothesized that HSD intake–induced elevated serum hippurate levels might be helpful in anti-PD1 therapy. To test our hypothesis, we subjected B16 melanoma-bearing mice to suboptimal (*so*) dose of anti-PD1 in combination with LSD, which have no protective effect on tumor growth. We found strong suppression (approximately 10-fold) of B16 melanoma when LSD was combined with anti-PD1 (*so*) that was associated with improved survival rate (Fig. 4, A to C). This suppression was accompanied with an inhibition of surface PD1 expression on NK cells in tumor-infiltrating immune cells, while no changes in LSD alone or anti-PD1 (*so*) alone group were observed (Fig. 4D). Furthermore, there was no significant changes in NK cell expression in combinatorial group, suggesting that LSD may act as an adjuvant whereby even slight increase in extracellular Na^+^ ion may enhance the efficacy of anti-PD1 immunotherapy (fig. S3E). Together, these data demonstrate that salt could act as an adjuvant to promote anti-PD1 immunotherapy for tumor regression.

### Gut microbiota is essential for HSD-mediated tumor immunity

**Fig. 5.**
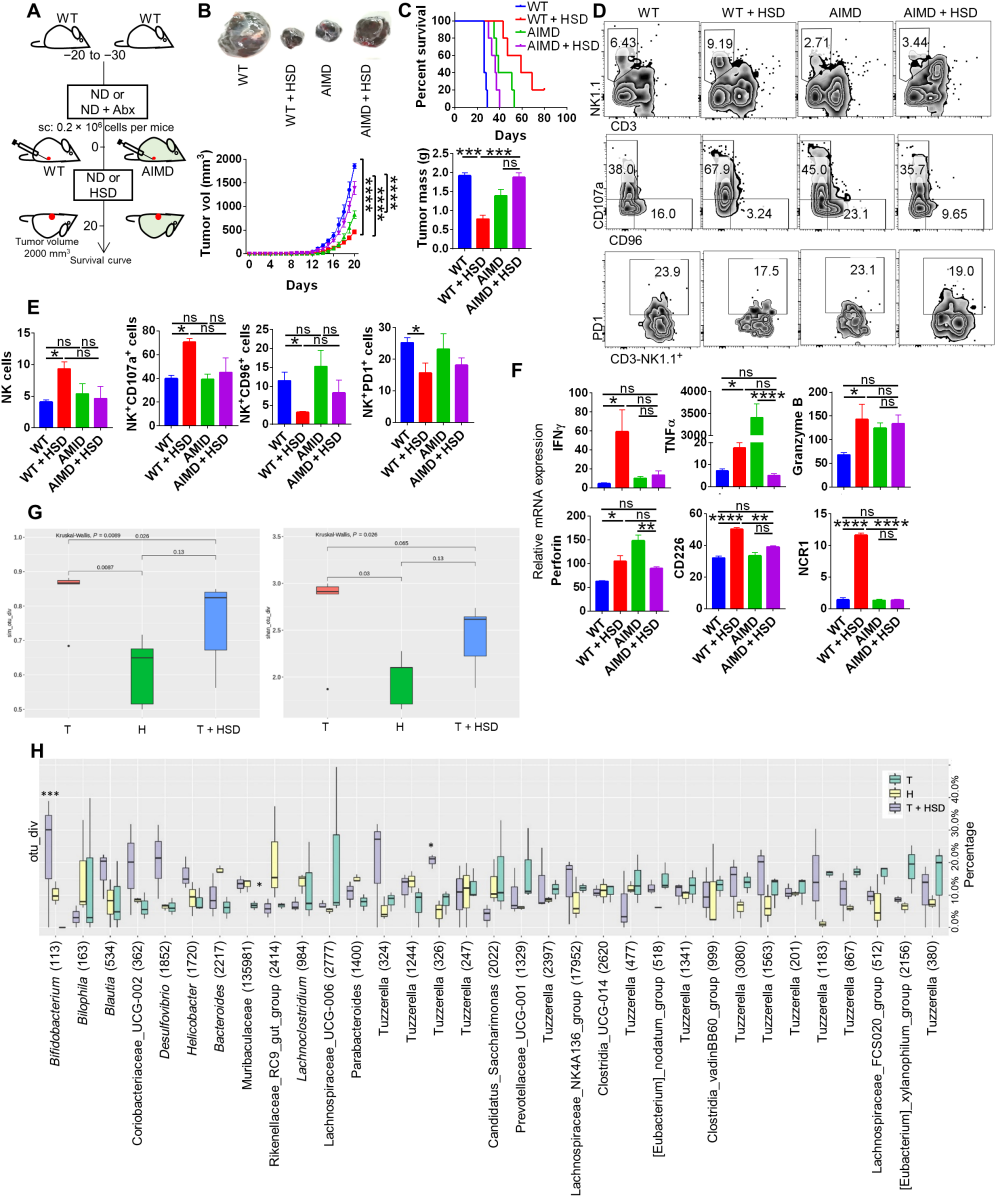
Changes in gut microbiota associated with HSD in B16 melanoma-bearing mice. The gut microbiota of the mouse was depleted by antibiotic regime, and then the effect of tumor progression was studied. (**A**) Schematic methodology for AIMD mice generation and comparison of (**B**) the progression of B16 melanoma in WT and AIMD mice in the presence or absence of HSD and (**C**) its survival. (**D** and **E**) Immunophenotyping results showing NK cells and associated markers along with (**F**) qPCR relative mRNA expression. The stool samples from the salt-fed mice were used for DNA isolation, and its 16*S* rRNA metagenomics analysis was carried out. (**G**) Simson and Shannon diversity plot indicating richness and evenness of the gut microbiota composition. (**H**) Plot showing OTU % age abundance of bacterial species in different salt groups. *P < 0.05, **P < 0.01, ***P < 0.001, and ****P < 0.0001 (Student’s *t* test or one-way ANOVA).

As indicated earlier, pHSD for 15 days was capable of a significant reduction in tumor progression. Immunophenotyping data of pHSD group showed elevated NK cell frequency in circulation and tumor-infiltrating immune cells as compared to the untreated group (fig. S4, A and B, respectively). Further, there was an up-regulation of CD107a expression and a down-regulation of CD96, PD1, and TIGIT expression on NK cells, suggesting that HSD imparts memory through factors that may be responsible for NK cell enrichment (Fig. S4B). Modulation in the gut microbiome is associated with tumor progression especially in patients receiving anti-PD1 therapy (*25*–*29*). Since HSD has been shown to modulate the gut microbiome in autoimmune settings (*5*, *30*), we investigated the role of gut microbiota in HSD-mediated tumor immunity. To do this, we generated mice in which their microbiome is depleted using antibiotics and hence referred as antibiotic-induced microbiome-depleted (AIMD) mice. Using AIMD mice, the effect of HSD on the progression of B16 melanoma was studied (Fig. 5A). Our data show that mice fed with HSD suppressed the tumor growth in microbiota competent wild type (WT) but not in AMID mice as measured by tumor volume and mass (Fig. 5B). We further studied the survival of B16-bearing WT and AMID mice and found that B16-bearing HSD-fed WT mice survived better than B16-bearing HSD-fed AMID mice (Fig. 5C). Further, flow cytometry analysis of tumor-infiltrating immune cells revealed an increased frequency of NK cells in HSD-fed WT mice; however, this pattern was abrogated in HSD-fed AMID mice [Fig. 5, D (top) and E]. Moreover, activation (CD107a) and inhibitory (CD96) markers of NK cells were found to be up- and down-regulated respectively in HSD-fed WT but not in HSD-fed AMID mice (Fig. 5D lower panel and 5E). The mRNA expression of NK cell–associated genes in AIMD mice was found to be down regulated or showed little or no change in HSD fed mice as compared to the AIMD mice fed with ND (Fig. 5F). Our data indicate that HSD might be modulating gut microbiota to induce tumor immunity, as ablation of gut microbiota results in loss of HSD-induced tumor immunity. It was reasoned that HSD-associated changes in gut microbiota diversity might be playing a role in NK cells activation and functions in tumor immunity. To identify the changes in gut microbiota, we carried out 16*S* ribosomal RNA (rRNA) metagenome analysis of stool samples from H, T, and T + HSD groups. The Simson and Shannon diversity plot indicated that there was an increase in both microbiota diversity richness and evenness in T + HSD group as compared to H and T groups (Fig. 5G). We prepared a detailed Shankey plot for T + HSD microbiome through operational taxonomic unit (OTU) quantitation that showed *Bifidobacterium* as one of the prominent bacterial population detected at species level (fig. S5). Moreover, OTU diversity plot showed prominent up-regulation of *Bifidobacterium* in T + HSD as compared to H group (Fig. 5H). Together, these data indicates a possibility of *Bifidobacterium* as a factor contributing toward HSD-mediated tumor immunity.

### FMT from HSD-fed mice results in tumor regression

**Fig. 6.**
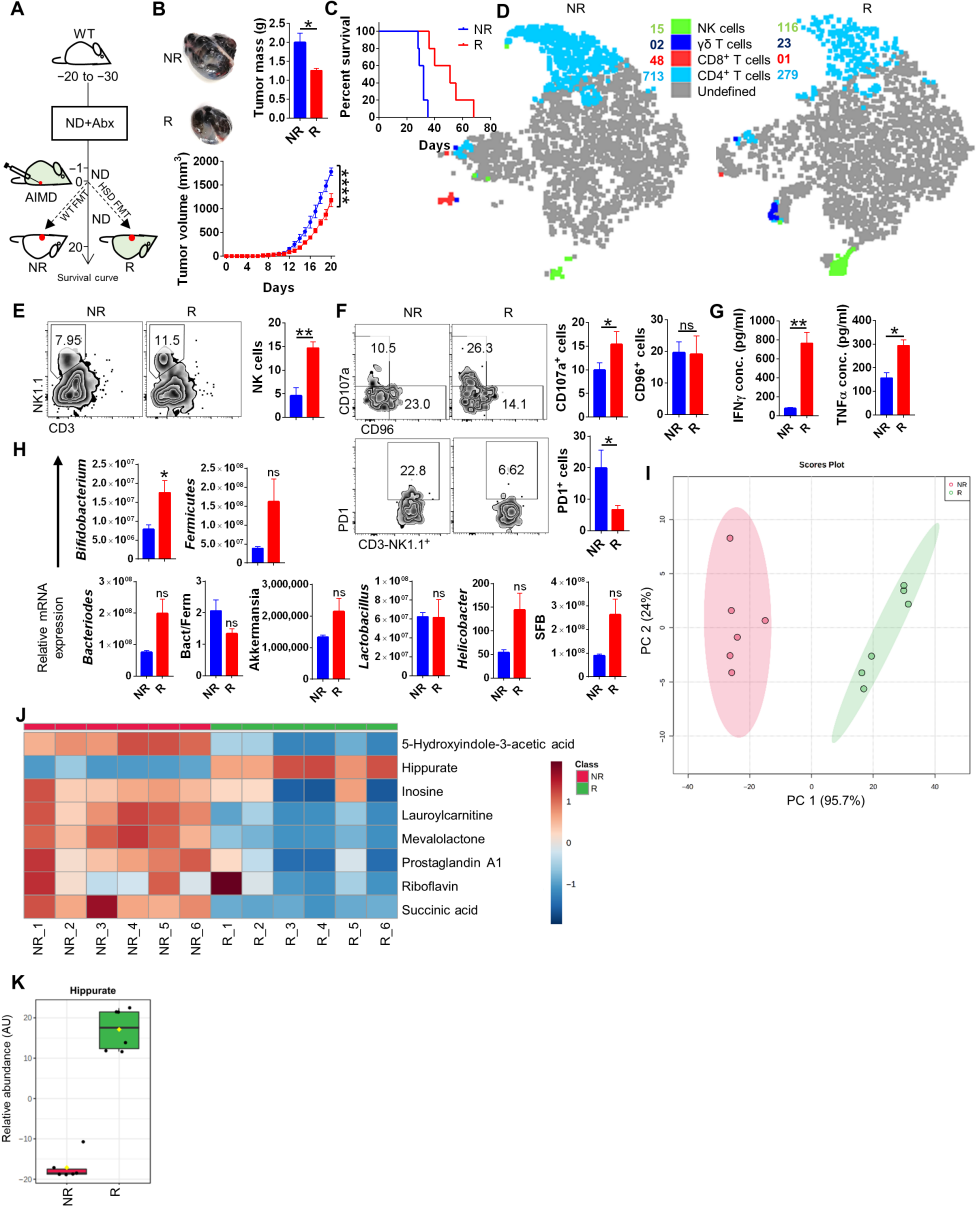
FMT from HSD donar to AIMD mice restores the gut levels of *Bifidobacterium.* Responder AIMD mice receiving FMT from HSD donar and NR AIMD mice receiving FMT from ND donar were injected with B16. (**A**) Schematic representation for (**B**) changes in melanoma progression and (**C**) survival curve; representative (**D**) tSNE plot depicting clusters of NK, γδT, CD4, and CD8 cells. (**E** and **F**) Immunophenotyping by FACS and (**G**) secreted cytokines expression. (**H**) Microbiota composition through qPCR. (**I**) PCA plot and (**J**) heatmap for serum metabolite profile through LC-MS and (**K**) levels of serum hippurate. *P < 0.05, **P < 0.01, and ****P < 0.0001 (Student’s *t* test or one-way ANOVA).

Our data suggested that HSD-induced tumor immunity requires gut microbiota; therefore, we hypothesized that HSD-mediated tumor immunity could be transferred through FMT from donor HSD-fed mice to the AIMD mice fed on ND. To do this, fecal material from mice fed with HSD were transferred through oral gavaging to AIMD mice. These mice were then implanted with B16 cells and were maintained on ND to monitor tumor growth (Fig. 6A). FMT from HSD mice (R) showed significant (~42%) regression of tumor volume and mass with improved survival as compared to AIMD mice receiving FMT from ND-fed mice (NR) (Fig. 6, B and C). The frequency of NK cells increased markedly in the R group as compared to the NR group in the tumor-infiltrating immune cells (Fig. 6, D and E). There was also an increase in NK cell function in R group as observed by increased CD107a expression and increased levels of IFNγ and TNFα (Fig. 6, F and G). Next, to understand the effect of FMT on the gut microbiome of R and NR mice, we carried out quantitative polymerase chain reaction (qPCR)–based profiling of stool samples for the enrichment of bacterial genus. Our qPCR data distinctly showed a 2.5-fold up-regulation of *Bifidobacterium* in the stool samples of R as compared to NR, while there were no significant changes observed in other prominent bacterial genera (Fig. 6H). This comprehensively pointed out that *Bifidobacterium* transfer through FMT to AIMD mice was able to restore NK cell frequency and tumor immunity. Since increased *Bifidobacterium* abundance in the gut was important for elevation in NK cell frequency, we reasoned that soluble factors secreted by *Bifidobacterium* into the circulation might be acting as mediators of this cross-talk. It has been previously reported that one of the by-products of the benzoate-mevalonate pathway is hippurate. Moreover, this benzoate-mevalonate pathway is an important metabolic pathway of *Bifidobacterium*. Therefore, it was reasoned that increased *Bifidobacterium* in the gut might be influencing the NK cell level and tumor immunity by secreted serum hippurate. Serum metabolites profile of R showed a significant increase in hippurate levels as compared to NR with distinct PCA distribution pattern (Fig. 6, I to K). Together, these data demonstrate that the modulation of gut microbiota by HSD enriches *Bifidobacterium*, which could be transfered to AIMD mice to provide tumor immunity through FMT.

### HSD results in increased gut permeability and intratumoral localization of *Bifidobacterium*

**Fig. 7.**
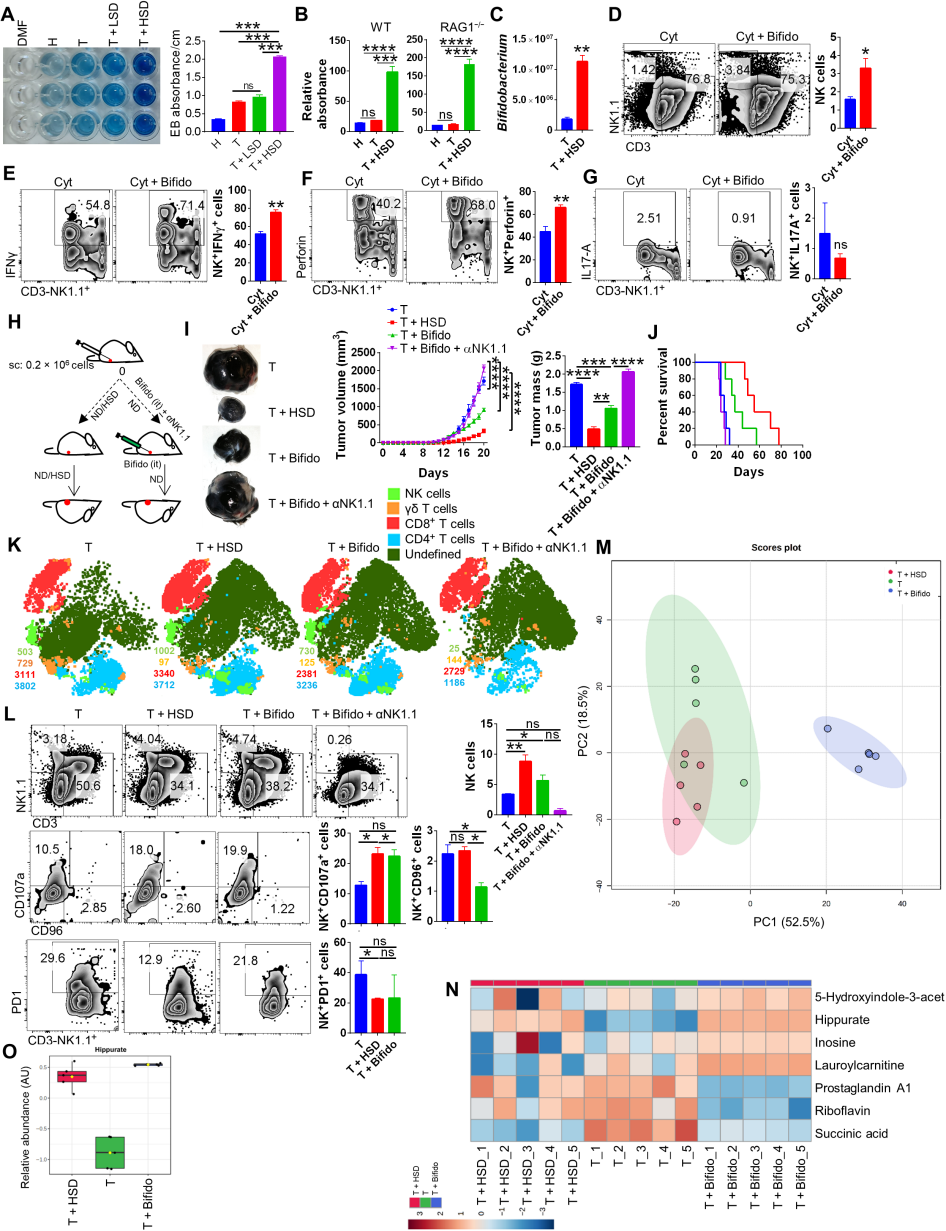
HSD increases the gut permeability causing *Bifidobacterium* intratumoral localization that boosts NK cells activation. (**A**) Elution of Evan’s blue dye from the colon of H, T, T + LSD, or T + HSD mice and its respective absorbance. (**B**) Relative absorbance from in situ FITC-dextran gut permeability assay for WT and RAG1^−/−^ mice. (**C**) qPCR relative quantiation for presence of *Bifidobacterium* in the tumor excised fom T or T + HSD mice. Splenocytes from healthy mice was cocultured in the presence or absence of increasing dose of *Bifidobacterium* and stimulated by NK cell–specific cytokines (IL-2 + IL-12). (**D**) Frequency of NK cells (**E** to **G**) and IFNγ, perforin, and IL-17A on NK cells was evaluated through flow cytometry. (**H**) Schematic representation for (**I**) tumor progression in mice with HSD or intratumoral *Bifidobacterium* administration with or without anti-NK1.1–neutralizing antibody treatment and (**J**) its survival curve. it, intratumoral. (**K**) Representative t-distributed stochastic neighbor embedding (t-SNE) plot depicting clusters of NK, γδT, CD4, and CD8 cells. (**L**) % age frequency of NK cells and associated surface molecules. (**M**) PCA plot for serum metabolomics profile of T, HSD, and T + *Bifidobacterium*. (**N**) Heatmap showing normalized levels of selected metabolites. (**O**) Relative abudance of serum hippurate levels in T, T + HSD, and T + Bifido condition. *P < 0.05, **P < 0.01, ***P < 0.001, and ****P < 0.0001 (Student’s *t* test or one-way ANOVA).

Several factors such as epigenetic, dietary, and disease status have been shown to regulate the permeability of the gut, which is important for the gut microbiota composition and integrity (*31*). It was, therefore, intriguing to understand the effect of HSD on gut permeability. To do this, mice were injected subcutaneously with B16 melanoma and fed with ND, LSD, or HSD, and gut permeability assay was performed using the Evan’s blue exclusion and fluorescein isothiocyanate (FITC)–dextran gut permeability assay. We found that there was ~2-fold increase in gut permeability in B16-bearing mice fed with HSD as compared to B16-bearing mice fed with LSD or tumor alone or healthy group (fig. S6A and Fig. 7A). We further tested the gut permeability in tumor bearing WT and RAG1^−/−^ mice and observed that HSD increases gut permeability of both B16-bearing RAG1^−/−^ and WT mice as compared to healthy control or B16-bearing mice fed with ND (Fig. 7B). These data, together, indicate a possibility of leaking out of gut bacteria due to increased gut permeability in T + HSD group. It was recently shown that there is an existence and colonization of bacteria in human cancers (*32*, *33*). On similar lines, it has been reported that there is intratumoral presence of *Bifidobacterium* (*33*). Our data suggest that HSD increases the abundance of *Bifidobacterium* and enhances gut permeability. This raises the possibility that gut *Bifidobacterium* may escape and colonize in the melanoma in the B16-bearing mice fed with HSD. To test this hypothesis, we carried out qPCR analysis for *Bifidobacterium* from tumor tissue in B16-bearing mice and B16-bearing mice fed with HSD. Strikingly, we found a ~6-fold increase in *Bifidobacterium* abundance within the melanoma of HSD-fed mice as compared to melanoma of ND-fed mice, suggesting the presence of *Bifidobacterium* in the melanoma tumor mass (Fig. 7C). These data further raise the possibility of direct cross-talk between NK cells and *Bifidobacterium*. To understand the effect of *Bifidobacterium* on NK cells, we cocultured the splenocytes in the presence or absence of NK-stimulating cytokine milieu (IL-2 + IL-12) with an increasing dose of *Bifidobacterium*, we found that in vitro coculture of *Bifidobacterium* with splenocytes was able to up-regulate the zone of the proliferation of NK cells and their frequency by two folds (Fig. 7D and fig. S6B). Further analysis revealed that *Bifidobacterium* increases the NK cell expression of IFNγ and perforin, but not IL-17A (Fig. 7, E to G). Quite interestingly, when *Bifidobacterium* was cocultured with splenocytes in presence of αCD3 (T cell stimulation), we also observed significant changes in IFNγ-, IL-17A, and perforin-expressing CD4^+^ and CD8^+^ T cells, without changes in their frequency, indicating that *Bifidobacterium* may also modulate T cell effector response (fig. S6C). These observations led us to hypothesize that introduction of *Bifidobacterium* in the tumor microenvironment may replicate in vitro effects of *Bifidobacterium* and may lead to NK cell up-regulation and tumor immunity even in the absence of HSD. We found that mice fed with ND receiving intratumoral injection of *Bifidobacterium* showed remarkable similarity in regression of tumor and overall survival along with increase NK cell response as compared to HSD-fed mice with no significant changes in other immune cell populations in tumor-infiltrating immune cells (Fig. 7, H to L). Moreover, when NK cells were depleted by anti-mouse NK1.1 antibody, the effect of *Bifidobacterium* administration was blunted (Fig. 7, H to L). We next analysed the serum metabolites profile of *Bifidobacterium*-injected mice and found that hippurate levels are significantly up-regulated upon colonization of *Bifidobacterium* within the tumor as compared to the B16-bearing tumor mice receiving no intratumoral *Bifidobacterium* (Fig. 7, M to O), thus providing evidence that cross-talk between *Bifidobacterium* and NK cells through hippurate is critical to tumor immunity mediated by HSD.

## DISCUSSION

Dietary components are identified as important environmental cues that influence human health and immunity (*1*–*3*, *34*). Excess dietary salt intake is linked to hypertension, cardiovascular complications, and more recently with tissue inflammation in inflammatory bowel disease (IBD), multiple sclerosis associated with enhanced T_H_17 cell response and inflammatory macrophages. Despite their effect in exacerbation of inflammatory diseases, the effects of salt, particularly Na^+^, on cancer immunotherapy has been a topic of great interest for research. In 1933, Shear (*35*) published the first comprehensive review summarizing the earlier findings and suggested that Na^+^ and K^+^ have modulatory potential on cancer cell growth in vitro. Recently, the protective effect of HSD on the tumor was provided in mouse model where salt was shown to inhibit tumor growth (*11*, *12*, *22*). It was shown that HSD modulated MDSC functions that overcome suppressive tumor microenviroment (*11*). However, cellular and molecular mechanisms that mediated tumor immunity induced by salt are poorly understood.

Hyponatremia is recognized as a common ionic imbalance in patients with cancers and was found to be associated with primary diagnosis (*18*, *19*). HSD intake may remove the hyponatremia condition and result in Na^+^ ion restoration that contribute to the tumor immunity. Our data in tumor-bearing mice show hyponatremia that was resolved with HSD and associated with enhanced tumor immunity. These findings were important in understanding potential factors which contribute to tumor immunity induced by HSD. Na^+^ is known to regulate diverse physiological functions; for example, it influences biological processes that can be sensed by immune cells and affect their differentiation and/or function in tissue inflammation. Since salt removes the immune-suppressive environment within tumor, we hypothesized that HSD-mediated tumor immunity may effectively work in various experimental tumor models. In line with this, our data showed that HSD-fed mice mount a robust regression of tumors of skin, lungs and breast. Our data further demonstrated that HSD effectively controls tumor growth post surgery.

Immune-profiling of TILs, draining lymph nodes (dLN), and spleen for six major immune cell populations viz. CD4^+^ T, CD8^+^ T, γδT, macrophages, MDSCs, and NK cells showed a distinct and significant enrichment of the NK cell population in B16-bearing HSD-fed mice. This increase in the frequency of NK cells was accompanied by up-regulation of activation markers and down-regulation of inhibitory markers on NK cells. The dominant role of NK cells in HSD-mediated tumor immunity was abrogated by depletion of NK cells in RAG1^−/−^ mice. Schlichter and MacCoubrey (*36*) in 1989 showed that low external Na^+^ ion concentration is inhibitory for NK cells cytotoxic activity and could be attenuated by increasing extracellular Na^+^ ion that results in Na^+^ influx inside NK cells. It could be reasoned that hyponatremia condition in cancer patients could be restored by HSD, which may enhance anti-tumor functions of NK cells.

Several shreds of evidence have now shown that the electrolytes balance and acid-base homeostasis controls multiple metabolic pathways, and therefore, any changes associated with ionic imbalance could perturb the metabolic pathways and lead to changes in the serum metabolites level (*37*). In line with this, our metabolomics profiling data show that metabolites associated with amino acid metabolism, Krebs’s cycle, and Warburg effect are significantly perturbed in HSD condition. Hippurate, a benzoic acid and mevalonic acid pathway derivative produced by the host and gut microbiota, showed a prominent increase in the serum of tumor-bearing mice kept on HSD. The elevated hippurate levels in the serum of HSD-fed tumor-bearing mice corroborated with the previously published report on Hippurate as a metabolic biomarker for the cancer patients that responded well to anti-PD1 immunotherapy (*21*). It is now known that although immunotherapy with antibodies against PD1, CTLA4, or PDL1 demonstrates significant efficacy, a significant number of cancer patients do not respond to immune-checkpoint therapy. Therefore, efforts have been made to identify factors (such as genetic, gut microbiota, and metabolic) that may predict the immune-responsiveness for checkpoint inhibitor therapy. In the above study, Hatae *et al.* (*21*) have shown that microbiome-derived hippuric acid is one of the seven identified clinical biomarker of a successful PD1 blockade immunotherapy in patients with cancer (R). In other words, this study provides the link between hippuric acid, anti-PD1 therapy and tumor regression. We found hippurate to be one of the metabolites, common between R and T + HSD serum metabolites profile. Since patients with higher hippurate responded well with anti-PD1 therapy, it was a possibility that increased hippurate levels by salt could help in reducing the dose of anti-PD1 antibody. Strikingly, suboptimal dose of anti-PD1 when combined with salt showed 10-fold decrease in tumor growth. It is also possible that salt, when combined with anti-PD1 therapy, may act as an adjuvant thereby directly enhancing the efficacy of the antibody therapy since hypotonic saline solution had been previously reported as an adjuvant in the treatment of acute haemorrhage condition (*38*). Another possibility is the involvement of serum hippurate, which is increased upon HSD diet. Spustovo *et al.* (*39*) in 1989 had shown a direct antitumor activity of hippurate in both in vitro and in vivo models. It is possible that low salt may also modulate serum hippurate levels, which could potentially induce antitumor immunity when combined with anti-PD1 therapy. In summary, we would like to emphasize that we are not sure what may be deriving this effect, and this remains one of the open-ended questions of our study.

In addition, elevated serum hippurate in HSD-fed B16 melanoma-bearing mice opened up the possibility of the involvement of gut microbiota in HSD-mediated tumor immunity, as hippurate is a secretory by-product of gut bacteria (*40*). Our data show that the effect of HSD was completely abrogated in AIMD mice, indicating the involvement of gut microbiota in HSD-mediated tumor immunity. HSD has been shown to modulate gut microbiota composition in the case of T_H_17 cell–mediated autoimmune disorders and is linked with decreased abundance of *Lactobacillus*, *Oscillibacter*, *Pseudoflaonifractor*, *Clostridium*, etc. (*5*, *30*). Our 16*S* rRNA metagenomics study revealed that HSD-fed mice showed an increase in both richness and evenness of microbiota composition as compared to the healthy control. This was an exciting finding as an increase in the composition of gut microbiota would mean significant changes in the abundance of the bacterial population. Our T + HSD metagenomics data showed a prominent increase in *Bifidobacterium* abundance. Whether an increase in Na^+^ levels in the circulation provides a suitable niche for colonization of *Bifidobacterium* or it inhibits the growth of other commensal bacteria, thereby promoting *Bifidobacterium* abundance, would be interesting to identify. Nonetheless, an increase in gut *Bifidobacterium* abundance by HSD strongly pointed at the crucial role of gut microbiota in HSD mediated-tumor immunity. An increased gut abundance of *Bifidobacterium* upon FMT was expected since we already showed that T + HSD feces has elevated *Bifidobacterium* levels; however, the findings that *Bifidobacterium* abundance was able to protect against melanoma and could lead to elevated NK cells frequency, and serum hippurate levels were interesting.

The influence of gut microbiota on immunological changes is well established (*1*, *4*). The resident commensal bacteria of the gut regulates immune homeostasis and are strongly correlated with IBD (*41*). *Bifidobacteria longum* and *Lactobacillus* species in the gut, for example, are involved in regulating the T_reg_ response through IL-10 production (*42*). It was shown that dietary probiotic supplementation of *Bifidobacterium* could directly enhance NK cell function in elderly subjects in a randomized control meta-analysis study (*43*). In line with the above trial, it could be asserted that there may be a cross-talk between *Bifidobacterium* and NK cells, which forms the basis of tumor immunity by HSD. Since elevated hippurate levels were observed in R group, we hypothesized that HSD causes an increase in gut *Bifidobacterium* that boosts the serum hippurate levels and promotes NK cell activation and enhances tumor immunity. Moreover, our data provide the first evidence that HSD increases gut permeability and thus facilitates dysbiosis of *Bifidobacterium*, which escapes the gut and colonizes the skin melanoma of the mice. Intratumoral localization of gut microbes has been described as a prominent feature of multiple human cancers, especially since several shreds of evidence now suggest that *Bifidobacterium* accumulation within the tumor may have beneficiary effects (*33*). Similarly, several studies on anti-PD1 immunotherapy in patients with cancers have now described *Bifidobacterium* as one of the prominent bacterial species of the gut responsible for successful anti-PD1 immunotherapy (*44*). Together, here, we provide the first evidence of a comprehensive cross-talk mechanism between *Bifidobacterium* and NK cells, which is mediated by hippurate to be the basis of HSD mediated tumor immunity. Our finding also opens up exciting therapeutic avenues for targeting cancer, one of which involves combining anti-PD1 immunotherapy with salt as an adjuvant. The other exciting finding of translational potential is the identification of *Bifidobacterium* as a crucial mediator of NK cell activity and tumor immunity. Through our findings, we propose that a consortium of microbiota mimicking HSD gut microbiota could have exciting translational values against cancer.

## MATERIALS AND METHODS

### Mice

C57BL/6, BALB/c, and RAG1^−/−^ mice were obtained from the Jackson Laboratory, housed, and maintained in a conventional pathogen-free environment at the small animal facility (SAF) at the Translational Health Science and Technology Institute (THSTI). Prior approval of the animal procedures was obtained from the institutional animal ethics committee (IAEC) (animal ethics approval number IAEC/THSTI/81), and all the experimental procedures involving animals were done in accordance with the guidelines laid by institutional animal ethics committee of THSTI.

### Cell lines

B16F10 melanoma cell line was obtained from American Type Culture Collection. LLC and luciferase-expressing 4T1 mammary carcinoma cell lines were gifts from A. Bajaj (Regional Centre Biotechnology, India). LLC and 4T1 cell lines were maintained in RPMI 1640 medium (Invitrogen) complete media containing 10% (v/v) fetal bovine serum (FBS) (Gibco), penicillin (100 U/ml; Sigma-Aldrich), streptomycin (100 μg/ml; Sigma-Aldrich), and 2 mM l-glutamine (Sigma-Aldrich). B16F10 cells were maintained in R10 complete media having RPMI 1640 complete media with 20 mM Hepes (Sigma-Aldrich). None of the cell lines used in this study were found in the Register of Misidentified Cell Lines maintained by the International Cell Line Authentication Committee (http://iclac.org/databases/cross-contaminations/).

### Syngeneic tumor models

Six- to 8-week-old C57BL/6 male mice were used for B16F10 and LLC injections, while 6- to 8-week-old BALB/c mice were used for 4T1 luciferase injections (*11*). Cells (0.2 million) were injected either subcutaneously (for melanoma) on the left flank region or were injected intravenously, and the animals were kept on ND, LSD, and HSD as specified in each experiment. Tumor volume was recorded each day until the end point according to the formula: volume (mm^3^) = *L* × *W* × *W*/2, where *L* is the length and *W* is the width of the tumor (in millimeters) along with changes in body weight, food, and water intake of the animals. Once tumors reached a volume of 1800 to 2000 mm^3^ (end point), animals were euthanized, and their samples were used for various study. In other set of experiment, the tumor animals were left for survival study, and the day of mortality of individual animal was recorded for percent survival curve for 80 days.

### Antibodies

Anti-mouse: α-CD45.2 (1:700; 104, BioLegend), α-CD3 (1:700; 145-2C11, BioLegend), α-CD4 (1:1000; GK1.5, BioLegend), α-CD8 (1:1000; 53-6.7, BioLegend), α-NK1.1 (1:700; PK136, BioLegend), α-γδTCR (1:700; GL3, BioLegend), α-PD1 (1:500; 29F.1A12, BioLegend), α–CTLA-4 (1:500; UC10-4B9, BioLegend; 1:500), α-TIM3 (1:600; B8.2C12, BioLegend), α-CD107a (1:700; 1D4B, BioLegend), α-CD96 (1:700; 3.3, BioLegend), α-TIGIT (1:700; 1G9, BioLegend), α-Gr1 (1:1500; RB6-8C5, BioLegend), α-CD11b (1:1500; M1/70, BioLegend), α-F4/80 (1:700; BM8, BioLegend), α-CD206 (1:700; C068C2, BioLegend), α-IFNγ (1:500; XMG1.2, BioLegend), α-TNFα (1:500; MP6-XT22, BioLegend), α–IL-17A (1:500; TC11-18H10, BioLegend), α-perforin (1:500; S16009A, BioLegend), and α-Ki67 (1:1000; SolA15, Thermo Fisher Scientific).

### Flow cytometry and intracellular cytokine staining

Processed cells from the spleen, lymph nodes, or tumor-infiltrating immune cells were stained for surface markers by fluorescence-tagged antibodies in fluorescence-activated cell sorting (FACS) buffer [phosphate-buffered saline (PBS) with 1% FBS] as previously described (*45*, *46*). For intracellular staining, cells were stained for surface molecules first followed by fixation and permeabilization (BD). For cytokine staining, cells were stimulated for 4 hours with phorbol 12-myristate13-aceate (PMA; 50 ng/ml; Sigma-Aldrich) and ionomycin (1 μg/ml; Sigma-Aldrich) in the presence of monensin (#554724 GolgiStop, BD Biosciences). Surface markers were stained first for 15 to 20 min at room temperature (RT), and then the cells were fixed in Cytofix and permeabilized with Perm/Wash Buffer using the Fixation Permeabilization Solution Kit (#554714, BD Biosciences). The Fc receptor were blocked with antimouse CD16/32 antibody incubation at RT for 20 min followed by staining with the fluorescenated antibodies for 20 min at RT in dark. The cells were then washed and analyzed by flow cytometry (Canto II, BD Bioscience). Data analysis was performed using FlowJo software (TreeStar).

### Quantitative polymerase chain reaction

RNA extraction from the samples were carried out by using an RNeasy kit (#74104, Qiagen), and complementary DNA (cDNA) were synthesized using an iScript cDNA Synthesis kit (#1708891, Bio-Rad). SYBR Green Gene Expression Assay using the Fast 7500 Dx qPCR System (Applied Biosystems) was then used for performing qPCR. *C*t values for the individual samples (genes) was then normalized to the endogenous control [β-actin/glyceraldehyde-3-phosphate dehydrogenase (GAPDH)] gene expression. All the primer sets were purchased from Sigma-Aldrich. The following primer sets were used (from Applied Biosystems; identifier in parentheses): Ifnγ (Mm01168134_m1), m_tnf_2 (NM_013693), and Gapdh (Mm99999915_g1). The qPCR results were analyzed using SDS 2.1 software. The *C*t value of the endogenous control gene was subtracted from the *C*t value of each target gene to determine the *C*t value change (ΔCt). The relative expression of each gene was then expressed as fold change calculated according to the previously used formula [POWER (2, -ΔCt) × 10,000] (*45*).

### Enzyme-linked immunosorbent assay

Cells isolated from different tissue samples (stimulated by using PMA and ionomycin as described above) or serum samples were used to perform sandwich enzyme-linked immunosorbent assay (ELISA) for mouse IFNγ or TNFα according to the previously described protocol (*45*). Briefly, ELISA plates were coated with α-IFNγ (1:250; R4-6A2, BioLegend) or α-TNFα (1:250; MP6-XT22, BioLegend) capture antibody in carbonate coating butter (100 μl per well) overnight at 4°C. The plate was then blocked with 3% milk powder in PBS. Thereafter, wells were washed with PBS + 0.05% Tween 20 (PBST) twice and incubated with samples (culture soup or serum samples) of 1:1 diluted in assay diluent (PBS + 1% milk powder) for 4 hours at RT. The wells were then washed thrice with PBST and incubated with biotinylated detection antibody α-IFNγ (1:1000; XMG1.2, BioLegend) and α-TNFα (1:1000; MP6-XT22, BioLegend) diluted in assay diluent at RT for 1 hour. The wells were then washed thrice with PBST and incubated with Avidin-HRP (Sigma-Aldrich) 1:10,000 dilution for 30 min at RT. Last, after washing it thrice with PBST, the plate was developed with trimethylboron substrate (50 μl) added in dark for 20 min at RT. The reaction was stopped with 3 M hydrochloric acid, and the absorbance of each well was measured at 550-nm wavelength in spectrophotometer. The corrected absorbance value was obtained by subtracting the absorbance of control samples.

### In vivo bioluminescence imaging for lung metastasis

In vivo bioluminescence (BLI) was performed as previously described for 4T1-injected BALB/c mice on day 16 (*47*). Briefly, animals were injected with d-luciferin (300 mg/kg) intraperitoneally and anesthetized after 20 min with isoflurane 2 to 3% that was reduced to 2% after the animlas were transferred to the imaging chamber. BLI in terms of photon emission per second was recorded from each mice by using intelligent visualization software (IVIS) imaging system (PerkinElmer Inc., Waltham, MA) at the optimal imaging time. The BLI data were quantitated by using IVIS software.

### Histology

Ten percent (v/v) formalin-fixed tissues were processed, stained with hematoxylin and eosin or Masson’s Fontana stain for melanin determination, or processed for immunohistochemistry by using α-Ki67 antibody (*48*). A certified histologist through blind sampling carried out the assessment of stained slides for the histological score on the scale of 0 to 5 (where 0 meant no staining).

### Isolation of tumor-infiltrating immune cells

At the end point of each study, the tumor were excised, and tumor-infiltrating immune cells were isolated by using Percoll density gradient as previously described with minor modifications (*17*). Briefly, tumor was dissociated by using a magnetic cell sorting dissociator (Miltenyi Biotec) in the presence of complete media in C-tube. The cells were pelleted at 400*g* for 6 min and then subjected to enzymatic dissociation in the presence of collagenase type IV (100 U/ml) and deoxyribonuclease I (100 μg/ml) in 2 ml of Hepes buffer containing CaCl_2_ (2.5 mg/ml) at 37°C for 1 hour. Dissociated tissues were then strained through 40-μm cell strainer and centrifuged at 400*g* for 10 min at RT. The supernatant was then slowly layered by 5 ml of 63% Percoll and then 3 ml of 47% Percoll mix followed by 2 ml of 33% Percoll. We centrifuged the gradient at 400*g* for 25 min without deacceleration. Last, tumor-infiltrating immune cells are collected as faint layer just above 63%, suspended in complete media, and used further.

### Depletion of NK cells

NK1.1^+^ cells in mice was depleted by giving intraperitoneal injection of 200 μg of monoclonal antibody (mAb) against NK1.1 (PK136; BioXcell) 24 hours before challenge, and after challenge, the antibodies were injected once weekly as previously described (*49*). Control group received intraperitoneal injection of isotype control antibody.

### IFNγ-neutralizing antibody

For neutralization of IFNγ cytokine, intraperitoneal injection of 200 μg of mAb against IFNγ (XMG1.2, BioXcell) was given twice weekly as previously described. Control group received intraperitoneal injection of isotype control antibody.

### Checkpoint immunotherapy by anti-PD1 inhibition

For the suboptimal in vivo inhibition of PD1 in mice, mAb (5 mg/kg) against PD-1 (RMP1-14, BioXcell) was injected intraperitoneally twice weekly starting from day 7 after B16 cell injection as previously described (*49*, *50*). Mice receiving suboptimal dose of PD1 were kept on either ND or LSD. Isotype control antibody was given to control groups.

### NK cell sorting from tumor-infiltrating immune cells

Sorting of NK cells from isolated tumor-infiltrating immune cells was carried out on FACSAria III (BD Biosciences) based on the surface markers CD3-NK1.1^+^ gating strategy. The purity of the sorted cells were ~95% in postsort analysis. Sorted NK cells were then used for in vitro qPCR assay.

### AIMD mice

AIMD was carried out in C57BL/6 mice according to the earlier published protocol (*51*). Amphotericin-B (0.1 mg/ml; Sigma-Aldrich) was administered orally for 3 days twice daily. From day 3 onward, mice were kept on ampicillin water (1 g/liter; Sigma-Aldrich) along with a cocktail of vancomycin (5 mg/ml; Sigma-Aldrich), metronidazole (10 mg/ml; Sigma-Aldrich), and neomycin (10 mg/ml; Sigma-Aldrich) given through oral gavage twice daily. Animals kept on antibiotics were housed in separate cages due to their caprophagy behavior. Depletion of microbiota was confirmed by absence of colony formation from stool samples upon culture.

### Fecal microbiota transplantation

Freshly excreted stool from the mice kept on ND or HSD were collected in 500 μl of sterile PBS directly from the anal orifice opening. The stool was then vortexed to achieve a homogenous suspension and snap-frozen at −80°C or used immediately for FMT in AIMD mice. One group of AIMD mice received 500 μl of FMT from mice kept on ND (NR), and the other group of mice received 500 μl of FMT from mice kept on HSD (R) through oral gavage for 3 days before the B16 injection. Mice receiving FMT were kept on ND throughout the experiment. The FMT protocol was adapted from previously published study with slight modification (*52*).

### 16*S* rRNA gene qPCR

Bacterial genomic DNA was isolated from mice fecal pellets using a PureLink microbiome DNA purification kit (#A29789, Thermo Fisher Scientific). qPCR was performed with universal 16*S* primer abundance and normalized to the endogenous control (rpoB/recA) for detecting bacterial. The primer sets used were as follows: Bacteroidetes (5′-GGARCATGTGGTTTAATTCGATGAT-3′ and 5′-AGCTGACGACAACCATGCAG-3′) Firmicutes (5′-GGAGYATGTGGTTTAATTCGAAGCA-3′ and 5′-AGCTGACGACAACCATGCAC-3′), *Bifidobacterium* [5′-TCGCGTC(C/T)GGTGTGAAAG-3′ and 5′-CCACATCCAGC(A/G)TCCAC-3′], *Lactobacillus* (5′-AGCAGTAGGGAATCTTCCA-3′ and 5′-CACCGCTACACATGGAG-3′), segmented filamentous bacteria (5′-GACGCTGAGGCATGAGAGCAT-3′ and 5′-GACGGCACGGATTGTTATTCA-3′), *Helicobacter* (5′-CTTAACCATAGAACTGCATTTGAAACTAC-3′ and 5′-GGTCGCCTTCGCAATGAGTA-3′), and *Akkermansia* (5′-CAGCACGTGAAGGTGGGGAC-3′ and 5′-CCTTGCGGTTGGCTTCAGAT-3′). The relative abundance of bacteria was calculated according to the “fold change” formula described above.

### 16*S* rRNA sequencing and metagenomics analysis

Extraction of genomic DNA was carried out by using a PureLink microbiome DNA purification kit (#A29789, Thermo Fisher Scientific) according to the manufacturer’s protocol. The samples were processed and analyzed by CoTeRi (NIBMG, India) for 16*S* rRNA sequencing using barcoded PCR primers targeting the V3-V4 region followed by libraries for its amplicon using a NEBNext Ultra DNA library preparation kit. These V3-V4 amplicon libraries were quantified and then loaded on the cBot for cluster generation and sequencing. PCR reactions were carried out in quadruplicate and pooled for sequencing on the Illumina HiSeq 2500 instrument, yielding 250–base pair paired-end sequence reads. 16*S* rRNA raw reads were quality-controlled with Trimmomatic version 0.39 (*53*). It removes adaptors and trims low-quality bases from the 3′ and 5′ end of reads to generate decontaminant raw reads and also discards less than 36 nucleotide-trimmed reads. A custom pipeline quantitative insights into microbial ecology (QIIME2, version 2018.11) was used to process and analyze decontaminant raw reads (*54*). DADA2 program was used to demux and join to generate long sequences. It processes the demultiplexed fastq files and produces sequence abundances. This was followed by grouping of all sequences as OTUs by pretrained Naive Bayes classifier (*55*), and their taxonomy were assigned by searching with Silva rRNA database release-138 at 97% sequence similarity.

### Alpha diversity indices

Shannon and Simpson diversity indices were computed and plotted through R scripts using the vegan, ape, ggplot2, phyloseq, and RAM packages. Differential taxon abundance analysis was also performed by RAM package of R script.

### Metabolomics analysis by LC-MS/MS

One hundred microliters of serum samples isolated from animals were mixed with LC-MS grade methanol at 1:3 ratio (serum:methanol), and the clear supernatant was transferred to a sterile LC vials (60 μl per tube). These samples were kept for drying (mi Vac Duo concentrator, Gene vac Ltd., UK). The dried pellet was then dissolved in 8:2 (v/v) acetonitrile:water for analysis on a chromatography system 5000 (Thermo Fisher Scientific). The separation was performed using an ultra performance liquid chromatography (UPLC) ultimate 3000 using the high strength silica (HSS) T3 column (2.1× 100 mm, 1.7 μm; Waters Corporation) at 40°C. The mobile phase was delivered at 300 μl/min and consisted of eluent A (water with 0.1% formic acid) and eluent B (acetonitrile with 0.1% formic acid), delivered in a gradient profile: 0 min, 1% B; 1 min, 15% B; 4 min, 35% B; 7 min, 95% B; 9 min, 95% B; 10 min, 1% B; 14 min, 1% B. The electrospray ionization source was operated for both positive mode (+) and negative (−) ionization at 120,000 resolution in MS1 mode and 30,000 resolution in data-dependent MS2 scan mode. The spray voltage used for these positive and negative modes is 4000 and 35,000 volts, respectively. Sheath gas and auxiliary gas were set to 42 and 11, respectively. The mass scan range was 50 to 1000 mass/charge ratio, AGC (automatic gain control) target was at 200,000 ions, maximum injection time was 80 ms for MS, AGC target was 20,000 ions, and maximum injection time 60 ms for MSMS was used.

### LC-MS/MS data processing and analysis

All acquired LCMS data were processed using the Progenesis QI for metabolomics (Water Corporation) software using the default setting. The untargeted workflow of Progenesis QI was used to perform retentiontime alignment, feature detection, deconvolution, and elemental composition prediction. Metascope plug of Progenesis QI was then used for the in-house library with accurate mass, fragmentation pattern, and retention time for database search. For further validation, online available spectral library was used. Peaks that had a coefficient of variation of less than 30% in pooled Quality control (QC) sample was kept for the further analysis of data. In addition, manual verification of each detected feature was done for the selection of right peaks. Analysis of data was done by using Metaboanalyst.ca online tool (*56*), which involves normalization, multivariate statistical analysis, and data annotation. The outlier samples were identified and removed by a combination of PCA and random forest followed by univariant analysis of variance (ANOVA).

### Evans blue in vitro permeability assay

The luminal content of the colon was washed using ice-cold PBS followed by injection of 200 μl of Evans blue solution [1.5% (w/v) in PBS] into the colon. Colon samples were then incubated in Krebs buffer (20 ml) at 5% CO_2_ incubator at 37°C for 30 min, washed with acetylcystine solution, then dried at 37°C for 1 day. The dry weight of colon was taken, and it was further incubated with 1 ml of dimethyl fumarate solvent at 55°C for another 24 hours. The absorbance was measured at 610 nm (*57*).

### FITC-dextran permeability assay

FITC-dextran (400 mg/kg; Sigma Aldrich; 4 kD) was orally administered to mice kept without food and water for 4 hours. Serum samples were obtained by retro-orbital bleeding after 4 hours after FITC-dextran administration. The serum fluorescence intensity was measured at an excitation wavelength of 490 nm and an emission wavelength of 530 nm using a spectrophotometer (*58*).

### Intratumoral quantitation of *Bifidobacterium*

For *Bifidobacterium* quantitation, tumor samples were sterilely collected and sprayed with 70% ethanol and homogenized immediately in 5 ml of sterile PBS containing 0.05% cysteine-HCl. Isolation of genomic DNA was done by a QIAamp PowerFecal DNA kit (QIAGEN, 12830–50) (*33*). Primer sets against *Bifidobacterium* (as mentioned above) was then used to determine the relative abundance of *Bifidobacterium* in the tumor samples through SYBR Green qPCR method as described above.

### In vitro stimulation

For in vitro activation of NK cell freshly isolated splenocytes were processed for single-cell suspension after red blood cell lysis and then seeded 0.1 million cells per well in 96-well plate followed by stimulation with IL-2 (1000 U) and IL-12 (10 ng/ml) for 48 to 72 hours (*59*, *60*). For the activation of T cells, wells of the 96-well plate was precoated with α-CD3 (2 μg/ml) for overnight at 4°C and then aspirated and seeded with splenocytes for 48 to 72 hours in 5% CO_2_ incubator (*45*). For studying the effect of salt on NK activation, 40 mM NaCl was added to the cytokine mixture (Cyt) containing IL-2 and IL-12 (*5*). For in vitro assay involving *Bifidobacterium*, the stimulated cells were grown in the presence or absence of *Bifidobacterium* at three different doses viz. 0.025 × 10^6^, 0.25 × 10^6^, or 2.5 × 10^6^ colony-forming units (CFU) per well (*33*). The proliferation of cells was observed under microscope, and the proliferated cells were then further used.

### In vivo supplementation of *Bifidobacterium*

For intratumoral inoculation, 2.5 × 10^6^ CFU *Bifidobacterium* (ProBiota Bifido, Seeking Health, WA, USA) was injection within tumor on days 7, 9, 11, and 13 after B16F10 injections in C57BL/6 mice according to the previously described protocol (*33*).

### Statistics

All the results were analyzed and plotted by using GraphPad prism 7.0 software. FACS, qPCR, ELISA, and metabolites studies were compared and analyzed by using one-way ANOVA or Student’s *t* test for *n* = 5 mice per group. Graphs are depicted as means with SEM. *P* value of less than 0.05 was considered as statistically significant.

## Notes

View/request a protocol for this paper from *Bio-protocol*.

## References

[1] A. L. Kau, P. P. Ahern, N. W. Griffin, A. L. Goodman, J. I. Gordon, Human nutrition, the gut microbiome, and immune system: Envisioning the future. Nature 474, 327–336 (2011). 21677749 10.1038/nature10213 PMC3298082

[2] K. M. Maslowski, C. R. Mackay, Diet, gut microbiota and immune responses. Nat. Immunol. 12, 5–9 (2011). 21169997 10.1038/ni0111-5

[3] S. P. Nobs, N. Zmora, E. Elinav, Nutrition regulates innate immunity in health and disease. Annu. Rev. Nutr. 40, 189–219 (2020). 32520640 10.1146/annurev-nutr-120919-094440

[4] L. Soldati, L. Di Renzo, E. Jirillo, P. A. Ascierto, F. M. Marincola, A. De Lorenzo, The influence of diet on anti-cancer immune responsiveness. J. Transl. Med. 16, 75 (2018). 29558948 10.1186/s12967-018-1448-0 PMC5859494

[5] N. Wilck, M. G. Matus, S. M. Kearney, S. W. Olesen, K. Forslund, H. Bartolomaeus, S. Haase, A. Mähler, A. Balogh, L. Markó, O. Vvedenskaya, F. H. Kleiner, D. Tsvetkov, L. Klug, P. I. Costea, S. Sunagawa, L. Maier, N. Rakova, V. Schatz, P. Neubert, C. Frätzer, A. Krannich, M. Gollasch, D. A. Grohme, B. F. Côrte-Real, R. G. Gerlach, M. Basic, A. Typas, C. Wu, J. M. Titze, J. Jantsch, M. Boschmann, R. Dechend, M. Kleinewietfeld, S. Kempa, P. Bork, R. A. Linker, E. J. Alm, D. N. Müller, Salt-responsive gut commensal modulates T_H_17 axis and disease. Nature 551, 585–589 (2017). 29143823 10.1038/nature24628 PMC6070150

[6] B. Yi, J. Titze, M. Rykova, M. Feuerecker, G. Vassilieva, I. Nichiporuk, G. Schelling, B. Morukov, A. Choukèr, Effects of dietary salt levels on monocytic cells and immune responses in healthy human subjects: A longitudinal study. Transl. Res. 166, 103–110 (2015). 25497276 10.1016/j.trsl.2014.11.007 PMC5538905

[7] W.-C. Zhang, X.-J. Zheng, L.-J. Du, J.-Y. Sun, Z.-X. Shen, C. Shi, S. Sun, Z. Zhang, X. Chen, M. Qin, X. Liu, J. Tao, L. Jia, H. Fan, B. Zhou, Y. Yu, H. Ying, L. Hui, X. Liu, X. Yi, X. Liu, L. Zhang, S.-Z. Duan, High salt primes a specific activation state of macrophages, M(Na). Cell Res. 25, 893–910 (2015). 26206316 10.1038/cr.2015.87 PMC4528058

[8] M. Kleinewietfeld, A. Manzel, J. Titze, H. Kvakan, N. Yosef, R. A. Linker, D. N. Muller, D. A. Hafler, Sodium chloride drives autoimmune disease by the induction of pathogenic T_H_17 cells. Nature 496, 518–522 (2013). 23467095 10.1038/nature11868 PMC3746493

[9] T. Morgan, J.-F. Aubert, H. Brunner, Interaction between sodium intake, angiotensin II, and blood pressure as a cause of cardiac hypertrophy. Am. J. Hypertens. 14, 914–920 (2001). 11587158 10.1016/s0895-7061(01)02135-5

[10] W.-C. Zhang, L.-J. Du, X.-J. Zheng, X.-Q. Chen, C. Shi, B.-Y. Chen, X.-N. Sun, C. Li, Y.-Y. Zhang, Y. Liu, H. Xiao, Q. Leng, X. Jiang, Z. Zhang, S. Sun, S.-Z. Duan, Elevated sodium chloride drives type I interferon signaling in macrophages and increases antiviral resistance. J. Biol. Chem. 293, 1030–1039 (2018). 29203528 10.1074/jbc.M117.805093 PMC5777245

[11] W. He, J. Xu, R. Mu, Q. Li, D. Lv, Z. Huang, J. Zhang, C. Wang, L. Dong, High-salt diet inhibits tumour growth in mice via regulating myeloid-derived suppressor cell differentiation. Nat. Commun. 11, 1732 (2020). 32265505 10.1038/s41467-020-15524-1 PMC7138858

[12] R. Willebrand, I. Hamad, L. Van Zeebroeck, M. Kiss, K. Bruderek, A. Geuzens, D. Swinnen, B. F. Côrte-Real, L. Markó, E. Lebegge, D. Laoui, J. Kemna, T. Kammertoens, S. Brandau, J. A. Van Ginderachter, M. Kleinewietfeld, High salt inhibits tumor growth by enhancing anti-tumor immunity. Front. Immunol. 10, 1141 (2019). 31214164 10.3389/fimmu.2019.01141 PMC6557976

[13] M. T. Orr, L. L. Lanier, Natural killer cell education and tolerance. Cell 142, 847–856 (2010). 20850008 10.1016/j.cell.2010.08.031 PMC2945212

[14] G. Alter, J. M. Malenfant, M. Altfeld, CD107a as a functional marker for the identification of natural killer cell activity. J. Immunol. Methods 294, 15–22 (2004). 15604012 10.1016/j.jim.2004.08.008

[15] C. J. Chan, L. Martinet, S. Gilfillan, F. Souza-Fonseca-Guimaraes, M. T. Chow, L. Town, D. S. Ritchie, M. Colonna, D. M. Andrews, M. J. Smyth, The receptors CD96 and CD226 oppose each other in the regulation of natural killer cell functions. Nat. Immunol. 15, 431–438 (2014). 24658051 10.1038/ni.2850

[16] S. Pesce, M. Greppi, F. Grossi, G. Del Zotto, L. Moretta, S. Sivori, C. Genova, E. Marcenaro, PD/1-PD-Ls checkpoint: Insight on the potential role of NK cells. Front. Immunol. 10, 1242 (2019). 31214193 10.3389/fimmu.2019.01242 PMC6557993

[17] Q. Zhang, J. Bi, X. Zheng, Y. Chen, H. Wang, W. Wu, Z. Wang, Q. Wu, H. Peng, H. Wei, R. Sun, Z. Tian, Blockade of the checkpoint receptor TIGIT prevents NK cell exhaustion and elicits potent anti-tumor immunity. Nat. Immunol. 19, 723–732 (2018). 29915296 10.1038/s41590-018-0132-0

[18] J. Jaal, T. Jõgi, A. Altraja, Small cell lung cancer patient with profound hyponatremia and acute neurological symptoms: An effective treatment with fludrocortisone. Case Rep. Oncol. Med. 2015, 286029 (2015). 26240768 10.1155/2015/286029 PMC4512576

[19] A. A. Onitilo, E. Kio, S. A. R. Doi, Tumor-related hyponatremia. Clin. Med. Res. 5, 228–237 (2007). 18086907 10.3121/cmr.2007.762 PMC2275758

[20] C. A. Harrison, D. Laubitz, C. L. Ohland, M. T. Midura-Kiela, K. Patil, D. G. Besselsen, D. R. Jamwal, C. Jobin, F. K. Ghishan, P. R. Kiela, Microbial dysbiosis associated with impaired intestinal Na^+^/H^+^ exchange accelerates and exacerbates colitis in ex-germ free mice. Mucosal Immunol. 11, 1329–1341 (2018). 29875400 10.1038/s41385-018-0035-2 PMC6162102

[21] R. Hatae, K. Chamoto, Y. H. Kim, K. Sonomura, K. Taneishi, S. Kawaguchi, H. Yoshida, H. Ozasa, Y. Sakamori, M. Akrami, S. Fagarasan, I. Masuda, Y. Okuno, F. Matsuda, T. Hirai, T. Honjo, Combination of host immune metabolic biomarkers for the PD-1 blockade cancer immunotherapy. JCI Insight 5, e133501 (2020). 31855576 10.1172/jci.insight.133501 PMC7098729

[22] Y. Xu, W. Wang, M. Wang, X. Liu, M.-H. Lee, M. Wang, H. Zhang, H. Li, W. Chen, High salt intake attenuates breast cancer metastasis to lung. J. Agric. Food Chem. 66, 3386–3392 (2018). 29553743 10.1021/acs.jafc.7b05923

[23] J. Lorenzen, C. E. Lewis, D. McCracken, E. Horak, M. Greenall, J. O. McGee, Human tumour-associated NK cells secrete increased amounts of interferon-gamma and interleukin-4. Br. J. Cancer 64, 457–462 (1991). 1911184 10.1038/bjc.1991.331 PMC1977624

[24] M. A. Curran, W. Montalvo, H. Yagita, J. P. Allison, PD-1 and CTLA-4 combination blockade expands infiltrating T cells and reduces regulatory T and myeloid cells within B16 melanoma tumors. Proc. Natl. Acad. Sci. U.S.A. 107, 4275–4280 (2010). 20160101 10.1073/pnas.0915174107 PMC2840093

[25] V. Gopalakrishnan, C. N. Spencer, L. Nezi, A. Reuben, M. C. Andrews, T. V. Karpinets, P. A. Prieto, D. Vicente, K. Hoffman, S. C. Wei, A. P. Cogdill, L. Zhao, C. W. Hudgens, D. S. Hutchinson, T. Manzo, M. P. de Macedo, T. Cotechini, T. Kumar, W. S. Chen, S. M. Reddy, R. S. Sloane, J. Galloway-Pena, H. Jiang, P. L. Chen, E. J. Shpall, K. Rezvani, A. M. Alousi, R. F. Chemaly, S. Shelburne, L. M. Vence, P. C. Okhuysen, V. B. Jensen, A. G. Swennes, F. McAllister, E. M. R. Sanchez, Y. Zhang, E. Le Chatelier, L. Zitvogel, N. Pons, J. L. Austin-Breneman, L. E. Haydu, E. M. Burton, J. M. Gardner, E. Sirmans, J. Hu, A. J. Lazar, T. Tsujikawa, A. Diab, H. Tawbi, I. C. Glitza, W. J. Hwu, S. P. Patel, S. E. Woodman, R. N. Amaria, M. A. Davies, J. E. Gershenwald, P. Hwu, J. E. Lee, J. Zhang, L. M. Coussens, Z. A. Cooper, P. A. Futreal, C. R. Daniel, N. J. Ajami, J. F. Petrosino, M. T. Tetzlaff, P. Sharma, J. P. Allison, R. R. Jenq, J. A. Wargo, Gut microbiome modulates response to anti–PD-1 immunotherapy in melanoma patients. Science 359, 97–103 (2018). 29097493 10.1126/science.aan4236 PMC5827966

[26] W. Li, Y. Deng, Q. Chu, P. Zhang, Gut microbiome and cancer immunotherapy. Cancer Lett. 447, 41–47 (2019). 30684593 10.1016/j.canlet.2019.01.015

[27] Y. Li, R. Tinoco, L. Elmén, I. Segota, Y. Xian, Y. Fujita, A. Sahu, R. Zarecki, K. Marie, Y. Feng, A. Khateb, D. T. Frederick, S. K. Ashkenazi, H. Kim, E. G. Perez, C.-P. Day, R. S. S. Muñoz, R. Schmaltz, S. Yooseph, M. A. Tam, T. Zhang, E. Avitan-Hersh, L. Tzur, S. Roizman, I. Boyango, G. Bar-Sela, A. Orian, R. J. Kaufman, M. Bosenberg, C. R. Goding, B. Baaten, M. P. Levesque, R. Dummer, K. Brown, G. Merlino, E. Ruppin, K. Flaherty, A. Ramer-Tait, T. Long, S. N. Peterson, L. M. Bradley, Z. A. Ronai, Gut microbiota dependent anti-tumor immunity restricts melanoma growth in *Rnf5*^−/−^ mice. Nat. Commun. 10, 1492 (2019). 30940817 10.1038/s41467-019-09525-y PMC6445090

[28] B. Routy, E. Le Chatelier, L. Derosa, C. P. M. Duong, M. T. Alou, R. Daillère, A. Fluckiger, M. Messaoudene, C. Rauber, M. P. Roberti, M. Fidelle, C. Flament, V. Poirier-Colame, P. Opolon, C. Klein, K. Iribarren, L. Mondragón, N. Jacquelot, B. Qu, G. Ferrere, C. Clémenson, L. Mezquita, J. R. Masip, C. Naltet, S. Brosseau, C. Kaderbhai, C. Richard, H. Rizvi, F. Levenez, N. Galleron, B. Quinquis, N. Pons, B. Ryffel, V. Minard-Colin, P. Gonin, J.-C. Soria, E. Deutsch, Y. Loriot, F. Ghiringhelli, G. Zalcman, F. Goldwasser, B. Escudier, M. D. Hellmann, A. Eggermont, D. Raoult, L. Albiges, G. Kroemer, L. Zitvogel, Gut microbiome influences efficacy of PD-1-based immunotherapy against epithelial tumors. Science 359, 91–97 (2018). 29097494 10.1126/science.aan3706

[29] S. Viaud, F. Saccheri, G. Mignot, T. Yamazaki, R. Daillère, D. Hannani, D. P. Enot, C. Pfirschke, C. Engblom, M. J. Pittet, A. Schlitzer, F. Ginhoux, L. Apetoh, E. Chachaty, P.-L. Woerther, G. Eberl, M. Bérard, C. Ecobichon, D. Clermont, C. Bizet, V. Gaboriau-Routhiau, N. Cerf-Bensussan, P. Opolon, N. Yessaad, E. Vivier, B. Ryffel, C. O. Elson, J. Doré, G. Kroemer, P. Lepage, I. G. Boneca, F. Ghiringhelli, L. Zitvogel, The intestinal microbiota modulates the anticancer immune effects of cyclophosphamide. Science 342, 971–976 (2013). 24264990 10.1126/science.1240537 PMC4048947

[30] A. Bier, T. Braun, R. Khasbab, A. Di Segni, E. Grossman, Y. Haberman, A. Leibowitz, A high salt diet modulates the gut microbiota and short chain fatty acids production in a salt-sensitive hypertension rat model. Nutrients 10, 1154 (2018). 30142973 10.3390/nu10091154 PMC6164908

[31] C. Chelakkot, J. Ghim, S. H. Ryu, Mechanisms regulating intestinal barrier integrity and its pathological implications. Exp. Mol. Med. 50, 1–9 (2018). 10.1038/s12276-018-0126-x PMC6095905 30115904

[32] D. Nejman, I. Livyatan, G. Fuks, N. Gavert, Y. Zwang, L. T. Geller, A. Rotter-Maskowitz, R. Weiser, G. Mallel, E. Gigi, A. Meltser, G. M. Douglas, I. Kamer, V. Gopalakrishnan, T. Dadosh, S. Levin-Zaidman, S. Avnet, T. Atlan, Z. A. Cooper, R. Arora, A. P. Cogdill, M. A. W. Khan, G. Ologun, Y. Bussi, A. Weinberger, M. Lotan-Pompan, O. Golani, G. Perry, M. Rokah, K. Bahar-Shany, E. A. Rozeman, C. U. Blank, A. Ronai, R. Shaoul, A. Amit, T. Dorfman, R. Kremer, Z. R. Cohen, S. Harnof, T. Siegal, E. Yehuda-Shnaidman, E. N. Gal-Yam, H. Shapira, N. Baldini, M. G. I. Langille, A. Ben-Nun, B. Kaufman, A. Nissan, T. Golan, M. Dadiani, K. Levanon, J. Bar, S. Yust-Katz, I. Barshack, D. S. Peeper, D. J. Raz, E. Segal, J. A. Wargo, J. Sandbank, N. Shental, R. Straussman, The human tumor microbiome is composed of tumor type–specific intracellular bacteria. Science 368, 973–980 (2020). 32467386 10.1126/science.aay9189 PMC7757858

[33] Y. Shi, W. Zheng, K. Yang, K. G. Harris, K. Ni, L. Xue, W. Lin, E. B. Chang, R. R. Weichselbaum, Y.-X. Fu, Intratumoral accumulation of gut microbiota facilitates CD47-based immunotherapy via STING signaling. J. Exp. Med. 217, e20192282 (2020). 32142585 10.1084/jem.20192282 PMC7201921

[34] D. C. Nieman, M. A. Lila, N. D. Gillitt, Immunometabolism: A multi-omics approach to interpreting the influence of exercise and diet on the immune system. Annu. Rev. Food Sci. Technol. 10, 341–363 (2019). 30633566 10.1146/annurev-food-032818-121316

[35] M. J. Shear, The rôle of sodium, potassium, calcium, and magnesium in cancer: A review. Am. J. Can. 18, 924–1024 (1933).

[36] L. C. Schlichter, I. C. MacCoubrey, Interactive effects of Na and K in killing by human natural killer cells. Exp. Cell Res. 184, 99–108 (1989). 2551707 10.1016/0014-4827(89)90368-6

[37] C. Menni, L. McCallum, M. Pietzner, J. Zierer, A. Aman, K. Suhre, R. P. Mohney, M. Mangino, N. Friedrich, T. D. Spector, S. Padmanabhan, Metabolomic profiling identifies novel associations with rlectrolyte and acid-base homeostatic patterns. Sci. Rep. 9, 15088 (2019). 31636301 10.1038/s41598-019-51492-3 PMC6803625

[38] J. Schavelzon, Hypotonic saline serum as an adjuvant in the treatment of acute hemorrhage. Prensa Med. Argent. 38, 2735–2737 (1951). 14883142

[39] V. Spustová, C. Oravec, Antitumor effect of hippurate. An experimental study using various mouse tumor strains. Neoplasma 36, 317–320 (1989). 2739810

[40] H. J. Lees, J. R. Swann, I. D. Wilson, J. K. Nicholson, E. Holmes, Hippurate: The natural history of a mammalian-microbial cometabolite. J. Proteome Res. 12, 1527–1546 (2013). 23342949 10.1021/pr300900b

[41] M. F. Neurath, Host–microbiota interactions in inflammatory bowel disease. Nat. Rev. Gastroenterol Hepatol. 17, 76–77 (2020). 31848474 10.1038/s41575-019-0248-1

[42] S. Sun, L. Luo, W. Liang, Q. Yin, J. Guo, A. M. Rush, Z. Lv, Q. Liang, M. A. Fischbach, J. L. Sonnenburg, D. Dodd, M. M. Davis, F. Wang, *Bifidobacterium* alters the gut microbiota and modulates the functional metabolism of T regulatory cells in the context of immune checkpoint blockade. Proc. Natl. Acad. Sci U.S.A 117, 27509–27515 (2020). 33077598 10.1073/pnas.1921223117 PMC7959554

[43] H. S. Gill, K. J. Rutherfurd, M. L. Cross, Dietary probiotic supplementation enhances natural killer cell activity in the elderly: An investigation of age-related immunological changes. J. Clin. Immunol. 21, 264–271 (2001). 11506196 10.1023/a:1010979225018

[44] A. Sivan, L. Corrales, N. Hubert, J. B. Williams, K. Aquino-Michaels, Z. M. Earley, F. W. Benyamin, Y. M. Lei, B. Jabri, M.-L. Alegre, E. B. Chang, T. F. Gajewski, Commensal *Bifidobacterium* promotes antitumor immunity and facilitates anti-PD-L1 efficacy. Science 350, 1084–1089 (2015). 26541606 10.1126/science.aac4255 PMC4873287

[45] S. Malik, S. Sadhu, S. Elesela, R. P. Pandey, A. S. Chawla, D. Sharma, L. Panda, D. Rathore, B. Ghosh, V. Ahuja, A. Awasthi, Transcription factor Foxo1 is essential for IL-9 induction in T helper cells. Nat. Commun. 8, 815 (2017). 28993609 10.1038/s41467-017-00674-6 PMC5634439

[46] S. Roy, A. Awasthi, ATP triggers human Th9 cell differentiation via nitric oxide-mediated mTOR-HIF1α pathway. Front. Immunol. 10, 1120 (2019). 31164892 10.3389/fimmu.2019.01120 PMC6536008

[47] J.-B. Kim, K. Urban, E. Cochran, S. Lee, A. Ang, B. Rice, A. Bata, K. Campbell, R. Coffee, A. Gorodinsky, Z. Lu, H. Zhou, T. K. Kishimoto, P. Lassota, non-invasive detection of a small number of bioluminescent cancer cells *in vivo*. PLOS ONE 5, e9364 (2010). 20186331 10.1371/journal.pone.0009364 PMC2826408

[48] Z. A. Rizvi, N. Puri, R. K. Saxena, Evidence of CD1d pathway of lipid antigen presentation in mouse primary lung epithelial cells and its up-regulation upon *Mycobacterium bovis* BCG infection. PLOS ONE 13, e0210116 (2018). 30596774 10.1371/journal.pone.0210116 PMC6312317

[49] T. A. Triplett, K. C. Garrison, N. Marshall, M. Donkor, J. Blazeck, C. Lamb, A. Qerqez, J. D. Dekker, Y. Tanno, W.-C. Lu, C. S. Karamitros, K. Ford, B. Tan, X. M. Zhang, K. McGovern, S. Coma, Y. Kumada, M. S. Yamany, E. Sentandreu, G. Fromm, S. Tiziani, T. H. Schreiber, M. Manfredi, L. I. R. Ehrlich, E. Stone, G. Georgiou, Reversal of indoleamine 2,3-dioxygenase–mediated cancer immune suppression by systemic kynurenine depletion with a therapeutic enzyme. Nat. Biotech. 36, 758–764 (2018). 10.1038/nbt.4180 PMC6078800 30010674

[50] M. T. Wolf, S. Ganguly, T. L. Wang, C. W. Anderson, K. Sadtler, R. Narain, C. Cherry, A. J. Parrillo, B. V. Park, G. Wang, F. Pan, S. Sukumar, D. M. Pardoll, J. H. Elisseeff, A biologic scaffold–associated type 2 immune microenvironment inhibits tumor formation and synergizes with checkpoint immunotherapy. Sci. Transl. Med. 11, eaat7973 (2019). 30700576 10.1126/scitranslmed.aat7973 PMC7254933

[51] A. Zarrinpar, A. Chaix, Z. Z. Xu, M. W. Chang, C. A. Marotz, A. Saghatelian, R. Knight, S. Panda, Antibiotic-induced microbiome depletion alters metabolic homeostasis by affecting gut signaling and colonic metabolism. Nat. Commun. 9, 2872 (2018). 30030441 10.1038/s41467-018-05336-9 PMC6054678

[52] S. M. Kim, J. R. DeFazio, S. K. Hyoju, K. Sangani, R. Keskey, M. A. Krezalek, N. N. Khodarev, N. Sangwan, S. Christley, K. G. Harris, A. Malik, A. Zaborin, R. Bouziat, D. R. Ranoa, M. Wiegerinck, J. D. Ernest, B. A. Shakhsheer, I. D. Fleming, R. R. Weichselbaum, D. A. Antonopoulos, J. A. Gilbert, L. B. Barreiro, O. Zaborina, B. Jabri, J. C. Alverdy, Fecal microbiota transplant rescues mice from human pathogen mediated sepsis by restoring systemic immunity. Nat. Commun. 11, 2354 (2020). 32393794 10.1038/s41467-020-15545-w PMC7214422

[53] A. M. Bolger, M. Lohse, B. Usadel, Trimmomatic: A flexible trimmer for Illumina sequence data. Bioinformatics 30, 2114–2120 (2014). 24695404 10.1093/bioinformatics/btu170 PMC4103590

[54] E. Bolyen, J. R. Rideout, M. R. Dillon, N. A. Bokulich, C. C. Abnet, G. A. Al-Ghalith, H. Alexander, E. J. Alm, M. Arumugam, F. Asnicar, Y. Bai, J. E. Bisanz, K. Bittinger, A. Brejnrod, C. J. Brislawn, C. T. Brown, B. J. Callahan, A. M. Caraballo-Rodríguez, J. Chase, E. K. Cope, R. Da Silva, C. Diener, P. C. Dorrestein, G. M. Douglas, D. M. Durall, C. Duvallet, C. F. Edwardson, M. Ernst, M. Estaki, J. Fouquier, J. M. Gauglitz, S. M. Gibbons, D. L. Gibson, A. Gonzalez, K. Gorlick, J. Guo, B. Hillmann, S. Holmes, H. Holste, C. Huttenhower, G. A. Huttley, S. Janssen, A. K. Jarmusch, L. Jiang, B. D. Kaehler, K. B. Kang, C. R. Keefe, P. Keim, S. T. Kelley, D. Knights, I. Koester, T. Kosciolek, J. Kreps, M. G. I. Langille, J. Lee, R. Ley, Y.-X. Liu, E. Loftfield, C. Lozupone, M. Maher, C. Marotz, B. D. Martin, D. McDonald, L. J. McIver, A. V. Melnik, J. L. Metcalf, S. C. Morgan, J. T. Morton, A. T. Naimey, J. A. Navas-Molina, L. F. Nothias, S. B. Orchanian, T. Pearson, S. L. Peoples, D. Petras, M. L. Preuss, E. Pruesse, L. B. Rasmussen, A. Rivers, M. S. Robeson, P. Rosenthal, N. Segata, M. Shaffer, A. Shiffer, R. Sinha, S. J. Song, J. R. Spear, A. D. Swafford, L. R. Thompson, P. J. Torres, P. Trinh, A. Tripathi, P. J. Turnbaugh, S. Ul-Hasan, J. J. J. van der Hooft, F. Vargas, Y. Vázquez-Baeza, E. Vogtmann, M. von Hippel, W. Walters, Y. Wan, M. Wang, J. Warren, K. C. Weber, C. H. D. Williamson, A. D. Willis, Z. Z. Xu, J. R. Zaneveld, Y. Zhang, Q. Zhu, R. Knight, J. G. Caporaso, Reproducible, interactive, scalable and extensible microbiome data science using QIIME 2. Nat. Biotech. 37, 852–857 (2019). 10.1038/s41587-019-0209-9 PMC7015180 31341288

[55] N. A. Bokulich, B. D. Kaehler, J. R. Rideout, M. Dillon, E. Bolyen, R. Knight, G. A. Huttley, J. Gregory Caporaso, Optimizing taxonomic classification of marker-gene amplicon sequences with QIIME 2’s q2-feature-classifier plugin. Microbiome 6, 90 (2018). 29773078 10.1186/s40168-018-0470-z PMC5956843

[56] J. Chong, D. S. Wishart, J. Xia, Using MetaboAnalyst 4.0 for Comprehensive and Integrative Metabolomics Data Analysis. Curr. Protoc. Bioinformatics 68, e86 (2019). 31756036 10.1002/cpbi.86

[57] Q. Mei, L. Diao, J. Xu, X. Liu, J. Jin, A protective effect of melatonin on intestinal permeability is induced by diclofenac via regulation of mitochondrial function in mice. Acta Pharmacol. Sin. 32, 495–502 (2011). 21441945 10.1038/aps.2010.225 PMC4001972

[58] J. Yang, I. Elbaz-Younes, C. Primo, D. Murungi, K. D. Hirschi, Intestinal permeability, digestive stability and oral bioavailability of dietary small RNAs. Sci. Rep. 8, 10253 (2018). 29980707 10.1038/s41598-018-28207-1 PMC6035168

[59] J. F. Clinthorne, E. Beli, D. M. Duriancik, E. M. Gardner, NK cell maturation and function in C57BL/6 mice are altered by caloric restriction. J. Immunol. 190, 712–722 (2013). 23241894 10.4049/jimmunol.1201837 PMC4080414

[60] S. Kim, K. Iizuka, H.-S. P. Kang, A. Dokun, A. R. French, S. Greco, W. M. Yokoyama, *In vivo* developmental stages in murine natural killer cell maturation. Nat. Immunol. 3, 523–528 (2002). 12006976 10.1038/ni796

