# Supplementary material for: High-salt diet mediates interplay between NK cells and gut microbiota to induce potent tumor immunity
Source: Sci Adv. 2021 Sep 10;7(37):eabg5016. doi: 10.1126/sciadv.abg5016 (PMC8442882; doi:10.1126/sciadv.abg5016)
Supplement: Supplementary file 1 — Figs. S1 to S6 [file sciadv.abg5016_sm.pdf]

Science Advances
AAAS

Supplementary Materials for

High-salt diet mediates interplay between NK cells and gut microbiota to induce potent tumor immunity

Zaigham Abbas Rizvi, Rajdeep Dalal, Srikanth Sadhu, Yashwant Kumar, Shakti Kumar, Sonu Kumar Gupta, Manas Ranjan Tripathy, Deepak Kumar Rathore, Amit Awasthi*

*Corresponding author. Email: aawasthi@thsti.res.in

Published 10 September 2021, *Sci. Adv.* **7**, eabg5016 (2021)
DOI: 10.1126/sciadv.abg5016

This PDF file includes:

Figs. S1 to S6

Supplementary figures

Fig. S1

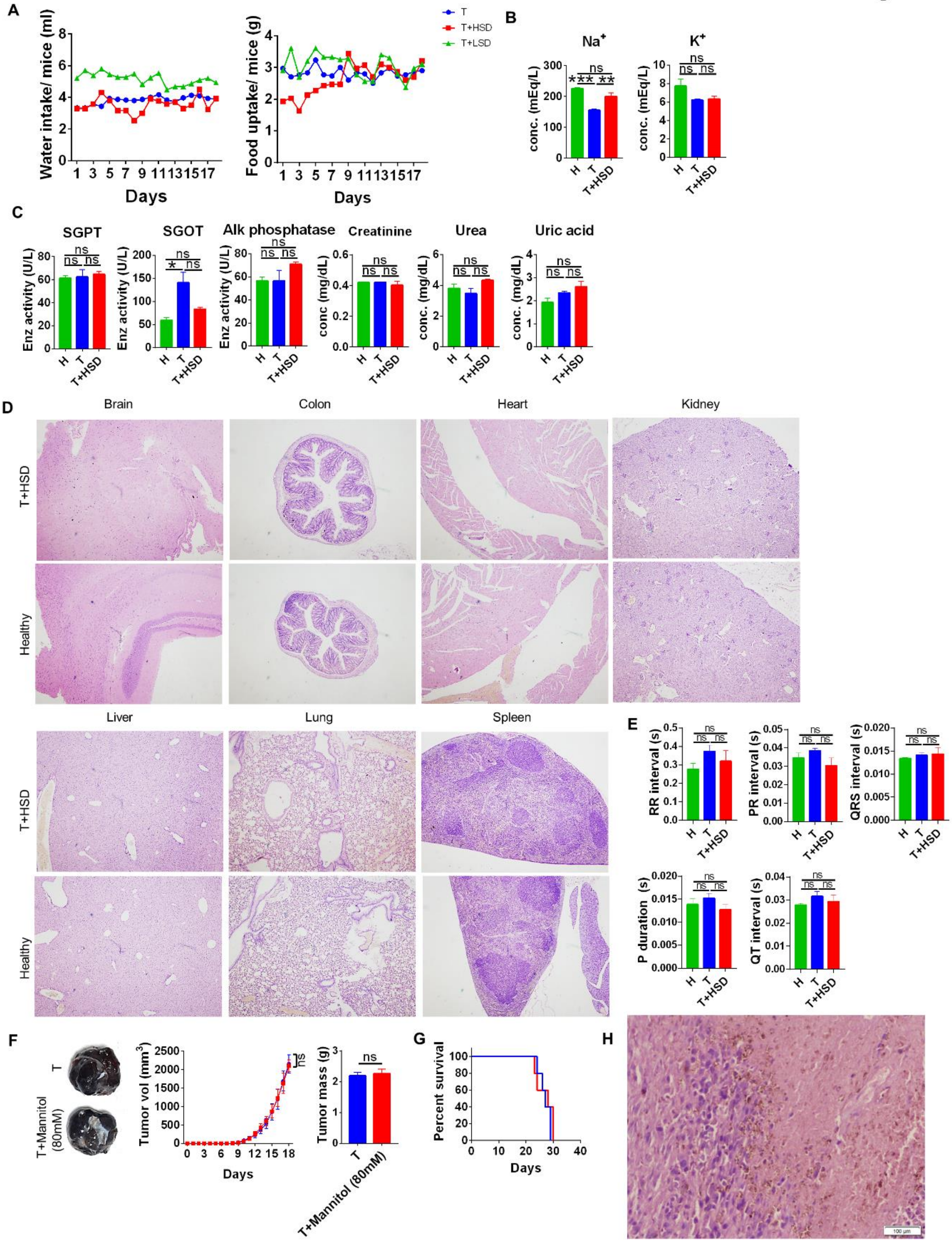

Fig. S1. HSD restores Na+ concentration in serum and do not exert any adverse effect. (A) Feeding behaviour of the mice. (B & C) concentration of serum electrolytes or liver and

kidney enzymes determined through biochemical methods (D) H&E stained sections from major organs showing no gross histological changes (E) ECG parameters for cardiac health (F) effect of equi-isoosmolar mannitol on B16 melanoma progression and (G) its survival. (H) anti-Ki67 minus antibody control for the IHC staining *P < 0.05 **P < 0.01, ***P < 0.001 (t-test or one-way anova).

Fig. S2

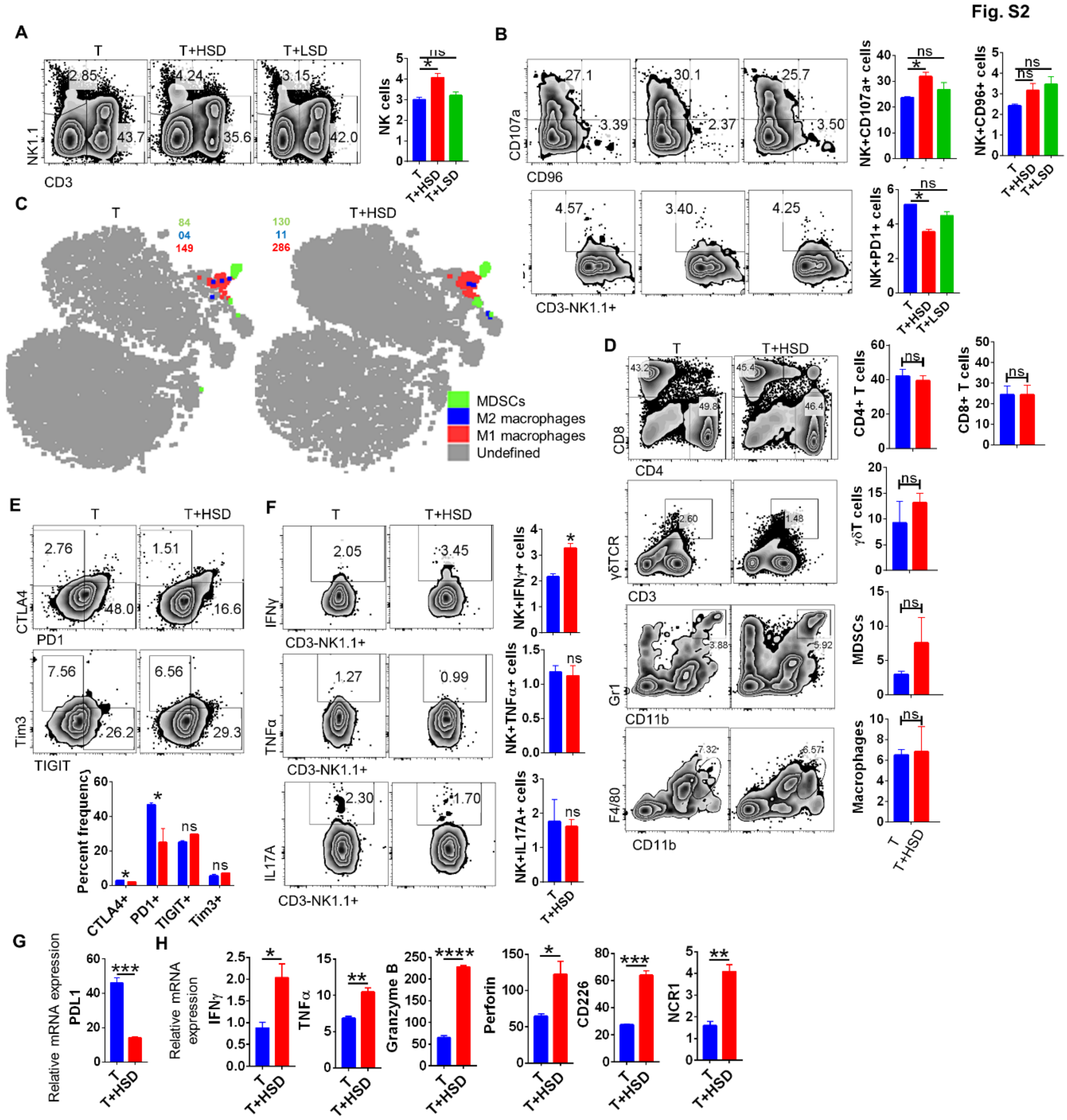

Fig. S2. Effect of HSD on major immune cell population in TILs, dLN and spleen in mice. Immunophenotyping of TILs, dLN and spleen were carried out to evaluate the frequency of major immune cell populations. Bar graph represents mean percent frequency $\pm$ SEM along with representative zebra plot. (A) NK cells frequency in the TILs (B) expression of CD107a, CD96 and PD1 on NK cells representative (C) tSNE plot depicting clusters of MDSCs, M1

and M2 macrophages (D) expression of CD4+, CD8+ T cells, γδT cells, MDSCs and macrophages (E) surface expression of inhibitory molecules (F) cytokine levels through intracellular cytokine staining (G) relative mRNA expression of PDL1 on cells isolated from skin melanoma. (H) relative mRNA expression of NK specific genes in sorted NK cells from TILs. $^*P < 0.05$, $^{**}P < 0.01$, $^{***}P < 0.001$, $^{****}P < 0.0001$ (t-test or one-way anova).

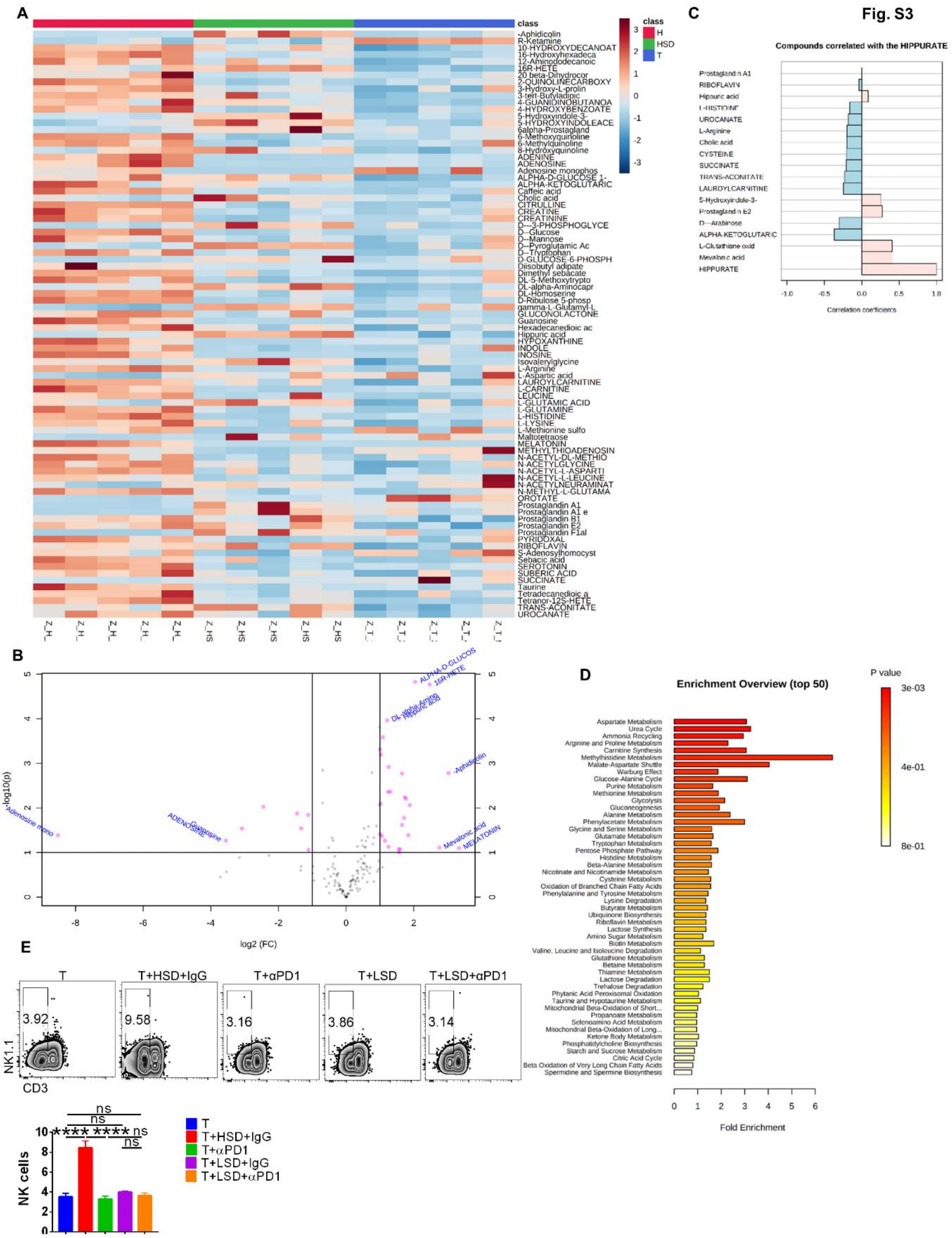

Fig. S3. Metabolomic profiling of salt fed mice. (A) Heatmap for all the significantly modulated metabolites, (B) correlation of other metabolites with Hippurate (C) volcano plot for metabolites under T and T+HSD condition (D) metabolic pathways significantly modulated (E) Percent frequency of NK cells in TILs isolated from mouse HSD alone, LSD alone, suboptimal dose of αPD1 alone or in combination with LSD. ****P < 0.0001 (one-way anova).

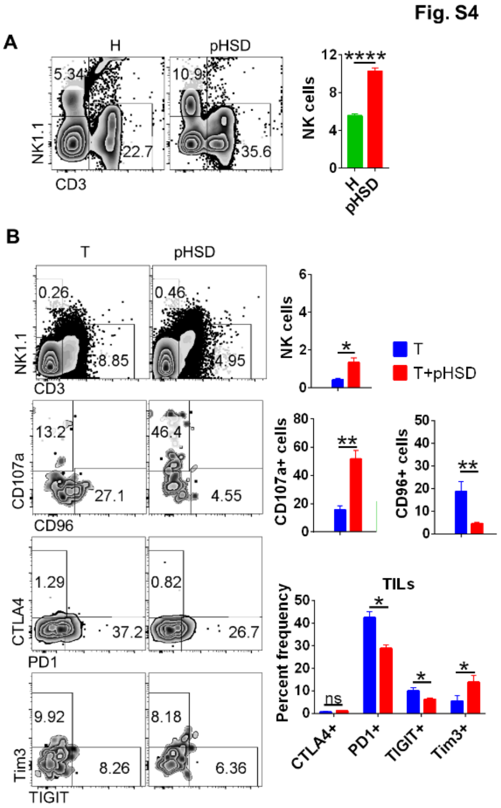

Fig. S4. Effect of pHSD on NK cell population. (A) Percent frequency of NK cells in the blood samples of H vs pHSD mice (B) %age positive cells for NK cells and its activation and inhibitory molecules in TILs isolated from mouse kept on pHSD for 15 days. Bar graph represents mean percent frequency ± SEM. *P < 0.05, **P < 0.01, ****P < 0.0001 (t-test or one-way anova).

Fig. S5

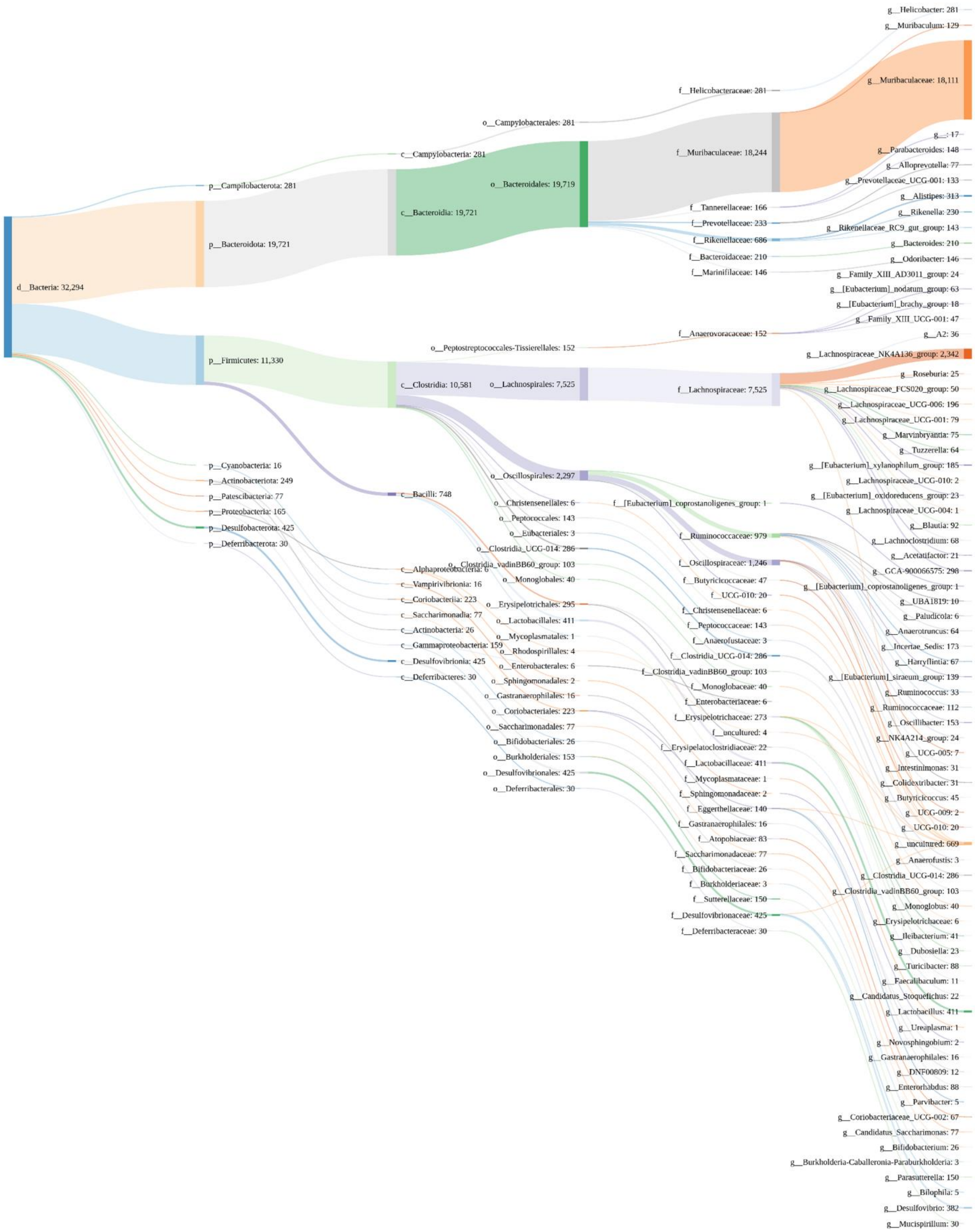

Fig. S5. Shankey plot for diversity of gut microbiota of HSD mice.

Fig. S6

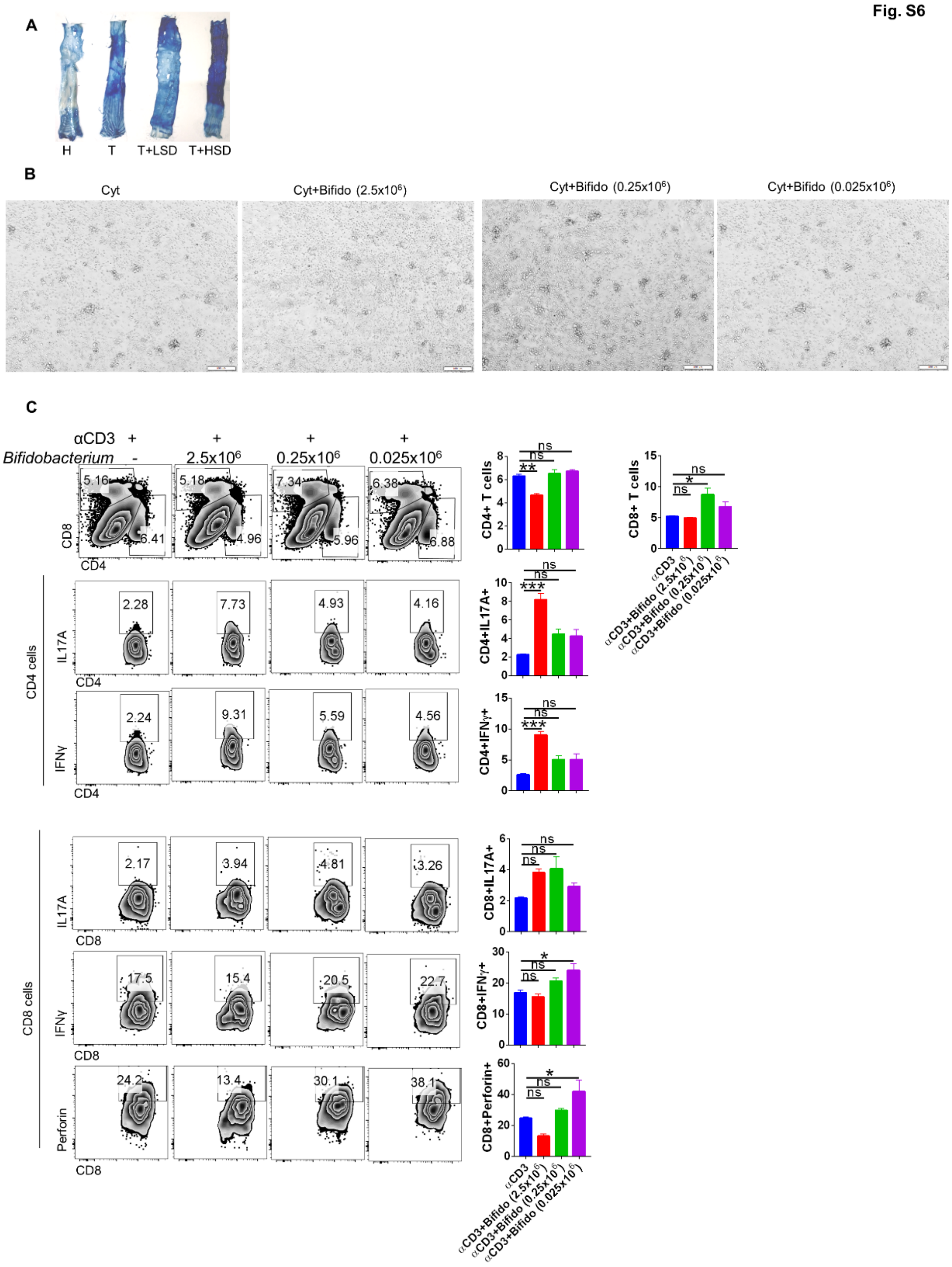

Fig. S6. Effect of HSD on gut permeability and in vitro stimulation of splenocytes by *Bifidobacterium*. (A) Gut permeability by evans blue exclusion method was determined and the eluded colon was imaged (B) microscopic images of splenocytes co-culture with *Bifidobacterium* in presence of IL2 and IL12 stimulation showing zone of NK cells proliferation (C) splenocytes co-cultured with *Bifidobacterium* was stimulated with anti-CD3 antibodies and immunophenotyping was carried out to see the changes in CD4+ and CD8+ T cells. *P < 0.05, **P < 0.01, ***P < 0.001 (one-way anova).
